# Finding polarized communities and tracking information diffusion on Twitter: a network approach on the Irish Abortion Referendum

**DOI:** 10.1098/rsos.240454

**Published:** 2025-01-15

**Authors:** Caroline B. Pena, Pádraig MacCarron, David J. P. O’Sullivan

**Affiliations:** ^1^ Mathematics Application Consortium for Science and Industry (MACSI), University of Limerick, Limerick, Ireland

**Keywords:** social network, Twitter, Information diffusion, polarization, community detection, text analysis

## Abstract

The analysis of social networks enables the understanding of social interactions, polarization of ideas and the spread of information, and therefore plays an important role in society. We use Twitter data—as it is a popular venue for the expression of opinion and dissemination of information—to identify opposing sides of a debate and, importantly, to observe how information spreads between these groups in our current polarized climate. To achieve this, we collected over 688 000 tweets from the Irish Abortion Referendum of 2018 to build a conversation network from users’ mentions with sentiment-based homophily. From this network, community detection methods allow us to isolate yes- or no-aligned supporters with high accuracy (90.9%). We supplement this by tracking how information cascades spread via over 31 000 retweet cascades. We found that very little information spread between polarized communities. This provides a valuable methodology for extracting and studying information diffusion on large networks by isolating ideologically polarized groups and exploring the propagation of information within and between these groups.

## 1. Introduction

Twitter—currently known as X^1^—has facilitated public debate about a variety of subjects, and as a result, it has received considerable attention from researchers who wish to gain insights into the relationships and mechanisms that govern social interactions [1]. As of August 2023, Twitter had 450 million monthly active users, making it the $14^{\text{th}}$ most popular social media platform in the world in terms of users [2]. In Ireland, by 2022, 1.35 million people—32.6% of the over-13-year-old audience—used Twitter [3].

Twitter is a popular venue for political discussions [4,5], such as referendums [6–9]. Ireland has a reasonably lengthy history of dealing with referendums [10], and two of them—the Irish Same-sex Marriage and the Irish Abortion Referendums—have received a lot of attention in social media, where millions of tweets were shared, and many public figures expressed their opinions [11,12].

Previous research by O’Sullivan *et al.* [9] examined the Irish Marriage Referendum and successfully used Twitter data and social network analysis to identify groups of users who were pro- and anti-same-sex marriage equality with a high degree of accuracy. Our research improves upon this work in two ways. First, we demonstrate that we can obtain better classification accuracy of users into polarized communities on two independent datasets (the Irish Marriage Referendum of 2015 and the Abortion Referendum of 2018) while using substantially less data, which is crucial given the cost of data gathering. Second, we extend the previous analysis by tracking not only how Yes and No supporters of the referendum interact individually but also how the information they share spreads across the network, within and between communities via the construction of retweet cascades [13–15], that is, a structure to describe who shared the same content from whom, following a timeline and relationships among users.

In summary, we address the following questions in this work:

- Is it possible to extend O’Sullivan *et al.*’s [9] analysis using different methods and less data (excluding the followership data)?
- How does information spread within and between ideological communities?

Key to our analysis is to show that users tend to group together according to the language they use in the discussion. The tendency that nodes have to associate preferentially with nodes that are similar to themselves in some way is a common phenomenon in many social networks, as in the classic school example, where children tend to form groups based on characteristics in common, like age [16]. This kind of selective linking is called *assortative mixing* or *homophily* in social networks. In the context of a referendum, where people either vote in favour or against it, we expect a polarized environment—a self-reinforcing system where users tend to talk to others who share the same opinions much more frequently than to those who share opposite ideas—and the presence of assortative mixing together with ideological label assignments is a good proxy for polarization [17]. In this sense, we will use a combination of methods to uncover assortative mixing and assign labels to the groups.

A combination of social-network structure and sentiment analyses has been used as a tool to understand and even predict human behaviour around many topics [18], such as stock market fluctuations [19,20], tracking the spread of viruses [21–25] and understanding the results of elections [26–28], and are powerful tools to uncover homophily [29–31]. In this work, we combine the analyses of sentiment and social structure to explore Twitter conversations about the Irish Marriage Referendum and the Irish Abortion Referendum.

In O’Sullivan *et al.* [9], a sentiment analysis tool [32] was used to quantify the positive and/or negative emotions of each tweet. This information was later aggregated to calculate the sentiment of each user in the network. The sentiment information was then used in conjunction with a combination of the conversation (who mentions whom in a tweet) and the followers (who are friends with whom on Twitter) networks to uncover community structure, which showed that the Irish Marriage Referendum was polarized with a large echo chamber effect—with a small group of users who communicated across community boundaries. This analysis relied heavily on the gathering of followership data, which is a time- and fund-consuming task. Therefore, our first aim in this work is to show that it is possible to use only the conversation graph—and not a combination of this and the followership graph—and sentiment to find communities of Yes and No supporters.

Once we have these groups, we extend previous work and give an example of the utility of the classification method, analysing the behaviour of cascade trees to understand how the community structure influences the spread of information in the social context. Cascade trees allow us to identify where the spread originated and to examine the structure it created. The link between community structure and how information spreads through a network has been reported to be a major factor in the spreading of content [33]. The spread of behaviour and information in social networks has been largely studied [34–36], as well as on a wide variety of social networks such as Facebook [37,38], Reddit [39], Google+ [40], TikTok [41] and LinkedIn [42], to name a few. We are particularly interested in how information spreads within and between communities in polarized conversation networks on Twitter.

To examine the online referendum’s social network structure, its interdependence with sentiment and the side in the debate that a user supports, we analyse two different datasets. One contains tracked tweets associated with the Repeal the 8^th^ Referendum (RT8; our focus in this article) and the second contains tracked tweets associated with the Irish Marriage Referendum—referred to as IMR (presented in appendix D). The Irish Marriage Referendum dataset is the same one utilized by O’Sullivan *et al.* [9].

In the remainder of the main text, we will explain our methodological approach (§2), followed by our case study on the Irish Abortion Referendum, where we show how we gathered the data and conducted a brief statistical analysis on it (§3.1), show how the network structure was created and how we incorporated sentiment scores into the network (§3.2), analyse the correlation between sentiment scores and the social structure (§3.3), which suggests the existence of polarized communities (§3.4). Using the polarized groups, we show our findings on the diffusion of information inside and between ideological communities (§3.5). The main part of this work ends with the conclusions around the Repeal the $8^{\text{th}}$ Referendum discussion and an outline of limitations and future work (§4).

## 2. Methodology

In this section, we provide a step-by-step guide for how the analysis will take place in the subsequent case study section. We start by collecting data. The type of data of interest is data that have a natural division of the individuals. Conversation data around controversial topics—for example, referendums or elections—are of this type. We gather tweets about the Irish Abortion Referendum of 2018 [43] and the Irish Marriage Referendum of 2015. The data must encompass different perspectives of the debate; therefore, the hashtags must be carefully selected, i.e. hashtags that are commonly used and informative of (and unique to, if possible) the matter of interest, at the same time, that they are representative of the different opinions from different sides of the conversation. As the focus is on the conversation that takes place between users, a conversation network must be built, where the nodes are the Twitter users in the collected dataset, and the links (edges) are mentions from one Twitter user to another regarding the matter being researched, where a mention represents one user either trying to engage another user in a dialogue, draw their attention to a piece of content (e.g. URL) or a reply to their tweet.

It is worth noting that most users will have a small number of tweets in the network, as they rarely interact with other users. The analysis must be made on the group of users who are highly active in the online discourse; therefore, we filter the network by only including a user if they have at least one mutual mention with other user(s). This results in a set of users who are more active than typical users on the topic and are more representative of those who engage with other users. This filtering has the added benefit of leaving us with a set of users who have more tweets on average to analyse the language that they use to express sentiment about the topic. Second, we maintain only the nodes that are in the largest strongly connected component of the network, which ensures that every node is connected to every other node, i.e. it is possible to reach any node in the network by starting from any random node.

A user’s sentiment represents how positive or negative their language is. Sentiment may be used as a proxy for homophily, i.e. users tend to group together according to their average sentiment (positive or negative). Therefore, one must first extract the sentiment from the collected tweets. We do this by using natural language processing to assign scores to each text content sent between pairs of users. We use a lexicon developed for short text that assigns negative scores to words that suggest negative language and positive scores to words that indicate positive language. The sentiment scores may vary considerably among tweets sent by a user, making this measurement noisy. Therefore, to extract a user's sentiment, we calculate the average sentiment of all tweets sent by the user.

Randomization tests may show sentiment alignment among users. If the results are not due to chance, sentiment can be used as a proxy for homophily. We analyse the correlation between the sentiment that a user sends to others and the sentiment they receive from others and test if users tend to connect to (mention) others that send the same type of sentiment (positive or negative) in the network. In this setting, it is reasonable to assume that there is a correlation between Yes and No supporters with positive and negative sentiment, respectively, as they will naturally use more positive or negative language to express their opinions about the referendum, i.e. a Yes supporter may use phrases like ‘I support this’, ‘I agree with it’, ‘this is great’, while a No supporter may say things like ‘I disagree with the proposal’, ‘this is not right’. However, in more complex settings where this might not be as clear, the network structure of who tends to talk to whom will help uncover groups of people who share similar opinions.

Therefore, to uncover the polarization structure, standard methods in graph theory that allow the identification of groups of nodes can be used. Community detection algorithms analyse the network topology and detect clustering patterns—groups of users that are highly connected among themselves and scarcely connected to nodes in another group—assigning each node a membership to a group of nodes. The community detection algorithms used in this work—fast greedy, Louvain, Leiden, spinglass, walktrap, leading eigenvector, infomap and label propagation—allow link weights to be used for the community detection. The link weights may increase the accuracy of the algorithm. Because sentiment alignment can be used as a proxy for homophily, as seen by previous randomization tests, and because nodes in the same group do tend to share similar characteristics, i.e. sentiment homophily, we assign the users’ average sentiment as link weights. However, weights cannot be negative. Therefore, we use the absolute value of users’ sentiment to determine the link weights in the network. This approach is consistent because users who express very negative sentiments are positioned far from those who express very positive sentiments in the network topology. We then choose the algorithm that performs the best based on modularity. The user classification is validated by annotated data, where we select a sample of the users. For annotation, the researcher is blinded to the username and community but classifies them as a Yes/No supporter based on all the tweets they sent to others regarding the studied referendum. Following this, error metrics can be calculated, such as balanced accuracy, to assess the method’s performance.

With the groups detected, one can then analyse the interactions of the users in light of the group labels. For example, we analyse how information spreads between and inside these groups. We do so by extracting the information diffusion cascades by using text and time information and the mentions network topology (the full dataset on mentions, without any filtering to avoid biases). We are then able to study who shares the same content from whom and add information on the membership from the community detection step to analyse how information spreads within and between polarized communities.

**Figure 1.**
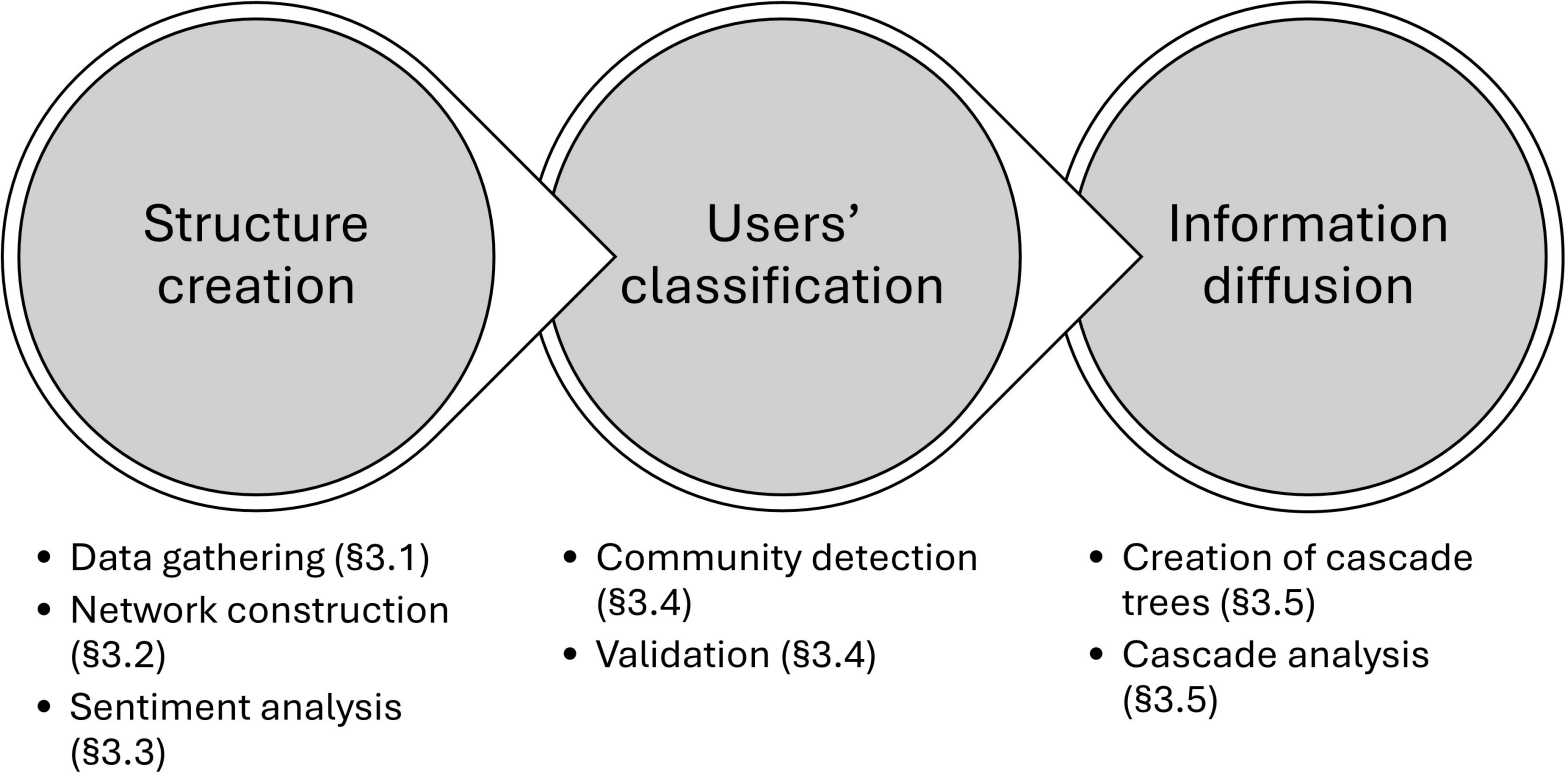
Schematic of our methodological approach.

In §3, we apply our method to the Twitter data for [43], explaining in detail the theory behind each step taken. Figure 1 illustrates our methodological approach.

## 3. Case study: the Irish Abortion Referendum of 2018

### 3.1. Background and data description

The referendum concerning abortion legislation, the so-called Repeal the 8^th^ Referendum, was one of the most recent referendums held in Ireland, on 25 May 2018. It abolished the 8^th^ Amendment of the Constitution—which gave the unborn equal rights to the mother and prohibited abortion in the State—and replaced it with the following wording [44]:

Provision may be made by law for the regulation of termination of pregnancies.

The Repeal the 8^th^ Referendum was one in a series of referendums about the matter held in Ireland since the inclusion of the 8^th^ Amendment in 1982 [45]. In 1992, two amendments were passed by referendums, which overturned the decisions preventing the right to travel for abortion and the right to distribute information about abortion services in other countries, and one amendment proposal—to exclude the risk of death by suicide as grounds for abortion—was rejected. In 2002, there was another attempt to exclude the risk of death by suicide as grounds for abortion, and also to create explicit criminal penalties for abortion. This bill did not pass either. The abortion discussion remained outside the mainstream of Irish politics until 2012, when Savita Halappanavar, an Indian dentist living in Ireland, had the termination of her pregnancy denied in an Irish hospital and died from complications arising from a septic miscarriage [45,46]. Protests and public discussion [46,47] culminated in the Repeal the 8^th^ Referendum in 2018. The turnout of this referendum was 64.13%, making it the fourth highest turnout for any referendum in the history of the State [45]. The ‘Yes’ side won by a margin of 66.4 to 33.6% [48]. Also, with 1429981 votes in favour, it received the biggest popular endorsement of any referendum question on abortion to date in the Irish State [45].

The tweet data on the Repeal the 8^th^ Referendum [43] was collected using the Twitter Academic Research API, which returns precise, complete and unbiased data from the Twitter archive [49]. However, as the data were collected a few years after the referendum, there could be missing tweets due to deletion or removal of the user’s account.

**Figure 2.**
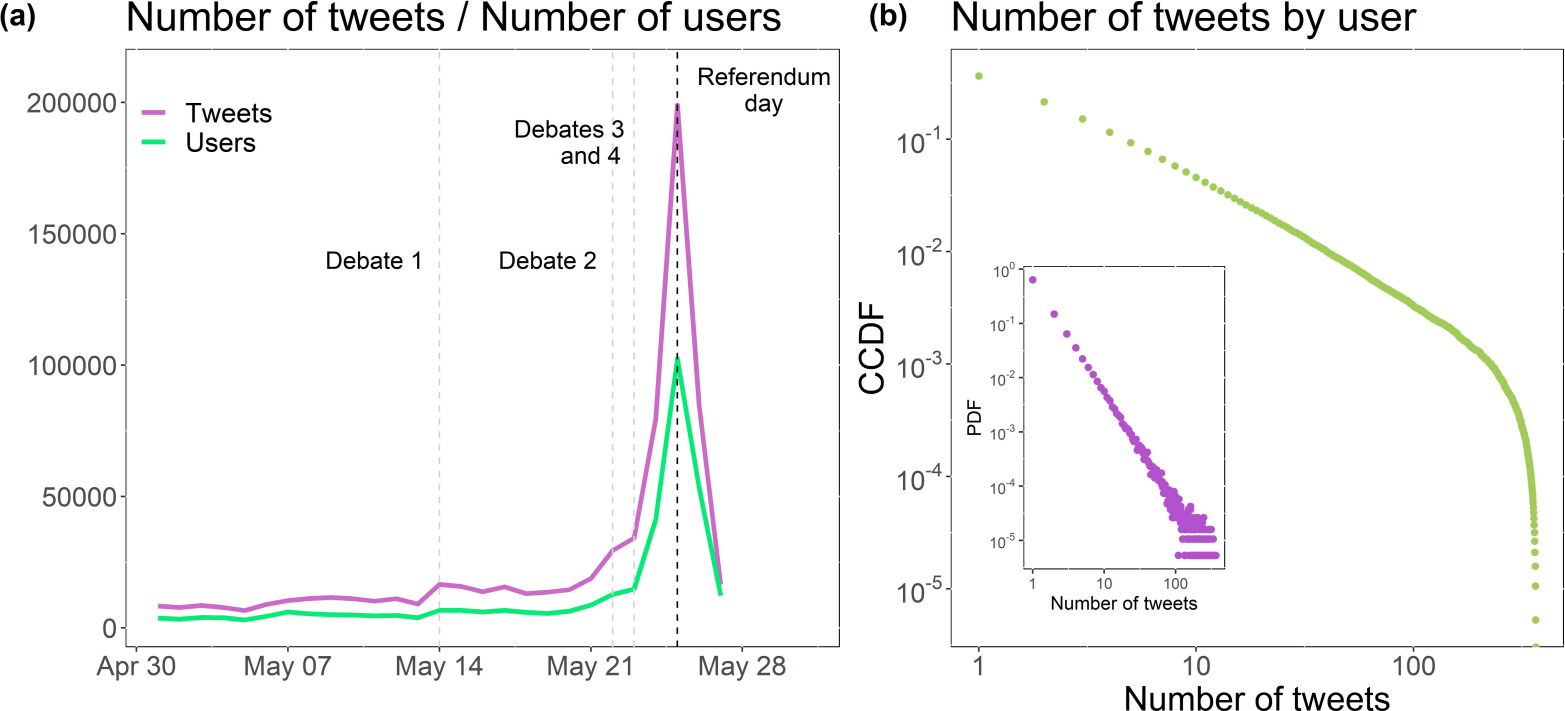
Data summary for the Repeal the 8^th^ discussion. (*a*) Repeal the 8^th^ activity time frame showing the televised debates and the day of the referendum. Note a high peak on the referendum day. (*b*) The complementary cumulative distribution function for the number of tweets per user and an inset of the probability distribution function of the same data.

For the Repeal the 8^th^ Referendum, every tweet containing the hashtags #repealthe8th, #savethe8th, #loveboth, #together4yes and #retainthe8th from 1 to 27 May 2018 (2 days after the referendum) was collected. Those hashtags in particular were chosen given their representativeness of both sides of the discussion and their popularity (see appendix B for more information on how we selected the hashtags). Four debates around the Repeal the 8^th^ discussion were televised, during which small increases in tweet numbers were recorded, and these can be seen in figure 2*a*. A large peak in the days leading up to referendum day is also observed. The data collected contains the text of the tweet, the date and time when the tweet was created, whether the tweet was a retweet or a reply (or neither, i.e. original), hashtags and mentions contained in the text. This information will be used to build the mentions network, which will be explained in §3.2, and to retrieve retweet cascades (§3.5).

Figure 2*b* shows that the number of tweets per user has a heavy-tailed distribution. The vast majority of users only posted a small number of tweets with the tracked hashtags, while a small number of users were responsible for a large volume of tweets. Out of the gathered tweets, 13.96% of the tweets were original posts, retweets represented 79.57% and replies represented 6.47% of the tweets (table 1). This shows that there was strong engagement among users in our dataset—the majority of tweets were retweets and replies—despite the heavy tail. The large proportion of retweets is indicative of the use of Twitter for information spread, which will be the focus of §3.5.

**Table 1.** Summary table of gathered dataset (RT8).

Attribute	Value
Period analysed	1−27 May 2018
No. of users	188 928
No. of tweets	688 903
No. of original tweets	96 182
No. of retweets	548 147
No. of replies	44 574

To analyse user behaviour around the topics in question, we need to understand how users connect and build the conversation network structure. The next section will explain how the network structure was created.

### 3.2. Network structure creation

Our goal is to construct a network that is reflective of the conversations taking place on Twitter around the Repeal the 8^th^ Referendum. For a conversation to take place, we require a network based on the mentions, that is, who talks to whom about the referendum. On Twitter, a mention to a user, text starting with the ‘@’ sign, can be generated by tagging the user in a tweet or by replying to the user (note: quoting tweets do not create explicit mentions as they do not create a link to the user that originally tweeted the content via an ‘@’ sign). We are interested in every form of mentioning among users. Therefore, we draw links connecting users by any type of mention (either tags or replies). It is also important to clarify that the mentions network is the observed communication between users rather than a structure underlying the possibility of connections. This is important in the next steps as we want to capture how users are connected and active in the network in order to detect polarized communities.

In this step, we keep 177324 unique users—11604 users did not mention anyone in our gathered tweets. Once we have the users (nodes) connected by who mentions whom, we want to filter the users who (i) are more active and engage with the subject [9,50–52], and will have more tweets to average the sentiment over, (ii) have strong social connections, which can be used to uncover community structures—politically active users tend to organize into homogeneous communities [53], which might be indicative of polarization. Therefore, we maintain only the nodes that have at least one mutual mention (i.e. A mentions B and B mentions A), creating the mutual mentions network (the network was downsized to 4967 nodes at this point). It is important to stress here that users do not necessarily have mutual mentions with all users they talk to; they only have to have mutual mention with one user in the network. We also filter the mutual mentions network into its largest strongly connected component, so that we have a network with active users who are strongly connected. The final mutual mentions network filtered by its largest strongly connected component has 3977 nodes.

Table 2 compares the largest strongly connected component of the full mentions network (the mentions network before filtering by the nodes with reciprocal mentions) with the largest strongly connected component of the mutual mentions network. It shows that the mutual mentions network contains users who have more connections (higher average out-degree) and are highly connected to each other (higher transitivity). Here, transitivity means that if a user mentions two other users, they also mention each other. It is important to highlight here that real data are known to be complex and noisy, therefore applying filters is a common practice in data analysis. Our aim is not to analyse the entire dataset originally scraped, but to build a concise network with only the most active and interconnected users to ensure that useful and clear results are generated. From here on, when we refer to the mentions network, we are referring to the largest strongly connected component of the mutual mentions network.

**Table 2.** Summary statistics for the full and the mutual mentions networks (RT8).

Network	Nodes	Links	Reciprocal links	Avg. out-degree	Transitivity
Full	8154	217 136	44 155	26.63	0.07
Mutual	3977	162 416	44 002	40.84	0.12

**Figure 3.**
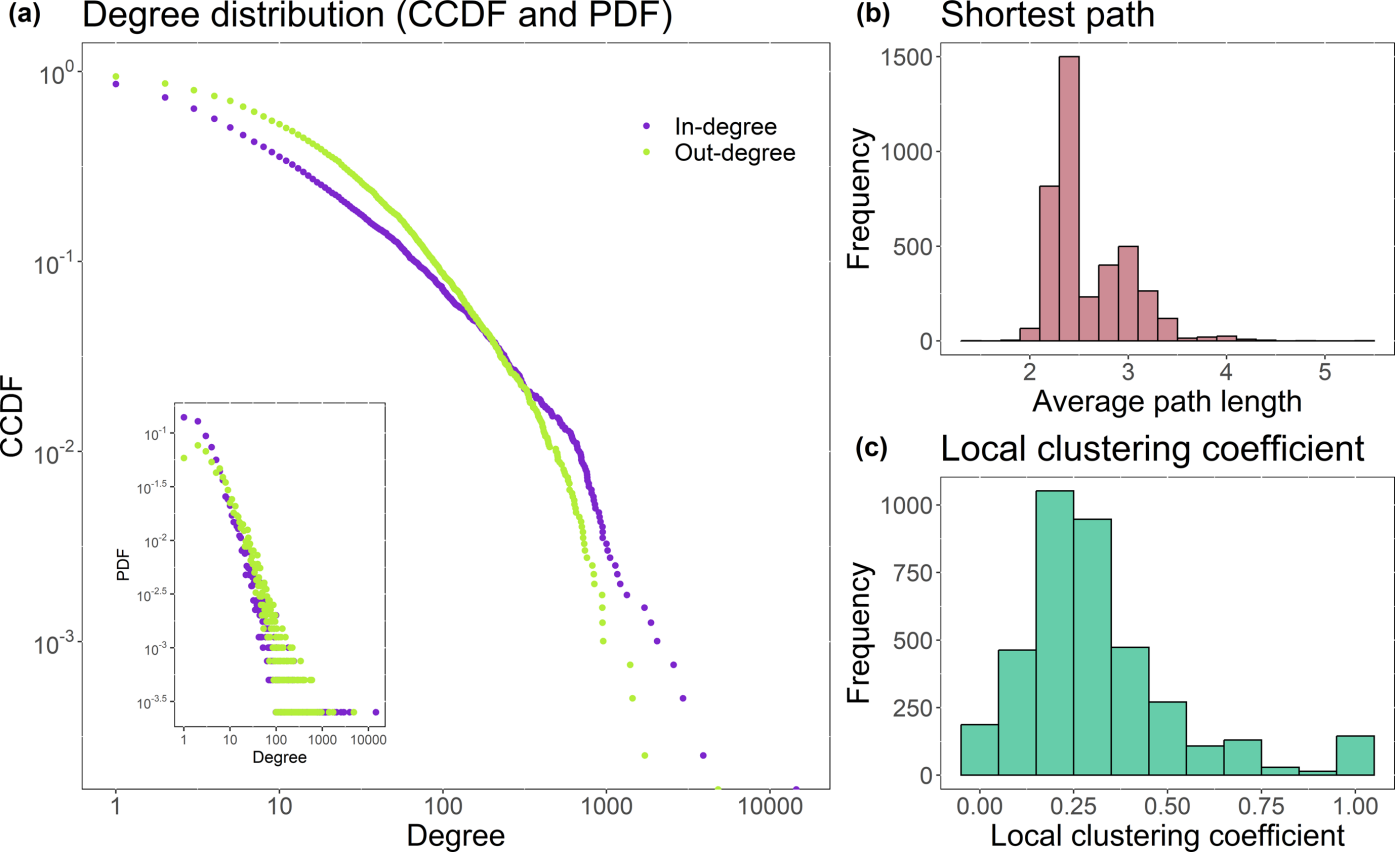
(*a*) Complementary cumulative distribution function (CCDF) for in-degree (purple) and out-degree (green) per user in the mutual network, and inset of the probability distribution function (PDF) of the same network; (*b*) average shortest path; (*c*) local clustering coefficients.

Figure 3*a* shows that, as expected for social networks [54], our mentions network has a heavy-tailed degree distribution. The curves show the complementary cumulative distribution function of the in-degree and the out-degree by user, with the inset showing the probability distribution function of our mentions network. A high number of users send (out-degree, green) a small number of tweets, while a small number of users post (and mention) a large volume of tweets. The same is observed for the quantity of tweets received (in-degree, purple) by each user.

The local clustering coefficient (figure 3*c*), suggests that the users are strongly interconnected. Almost 20% of the users have a local clustering coefficient greater than 0.5, or in other words, almost 20% of the users have neighbours with a probability greater than 50% of being connected together.

Figure 3*b* shows the mean shortest distance between vertex pairs. The spread of information is faster in networks that have a small average path length [16]: if it takes only a small number of steps for information to spread from one person to another, then the information will propagate much faster compared with a network with a large average number of steps between nodes. The most frequent average path length between nodes observed in our mutual network for the RT8 data is 2–3 steps, suggesting that the spread of information occurs quickly among users, which will be further explored in §3.5.

#### 3.2.1. Sentiment scores

Emotions are important in communication, and large-scale studies of communications need methods to detect emotions [18]. For this purpose, various sentiment lexicons have been developed in the past few years in an attempt to better characterize and treat sentiment from texts [32,55–60]. Sentiment analysis has been used as a tool to understand and even predict human behaviour around many topics [18], such as financial markets [19,20], tracking the spread of viruses [21–25], film box-office performance [61–63], reviews [64] and even the analysis of elections [26–28] and storm impacts [65], and more recently the effects of COVID-19 outbreaks on mental health [66,67]. Also, recent studies have successfully pointed out how sentiment can be used to help model the spread of information on Twitter [9,56,68].

While O’Sullivan *et al*. [9] made use of *SentiStrength* [32] and its built-in lexicon for sentiment analysis, we chose to use the R package *tidytext* [69] in this study due to its usability and easy implementation with large amounts of text. The lexicons in the *tidytext* package are based on unigrams, i.e. single words, meaning the respective algorithms check word by word to assign scores. We use *AFINN* [70], a lexicon constructed specifically for microblogs, therefore appropriate to analyse tweets. Furthermore, *AFINN* performs well in polarization analyses on Twitter, according to Hernández *et al*. [71]. It assigns the words a score that ranges between −5 and +5, with negative scores indicating negative sentiment and positive scores indicating positive sentiment (see the illustrative example in figure 13).

The sum of positive and the sum of negative scores was then obtained for each tweet. Finally, the positive scores were rescaled in order to fall into the interval from 0 to 5, and the same was done for the negative scores, with the range of scores to be between −5 and 0, instead. The final sentiment score for a tweet is the sum of its negative and positive scores. Tweets containing neither positive nor negative scores are assigned a sentiment score equal to zero (neutral). Figure 13 in appendix A exemplifies how the mentions network was built. On the network topology, each mention tweet between two users is represented as a link in the mentions network, and each one of those links now has an attribute containing the sentiment score of its text. In the next section, we will use those sentiment scores and the network structure to understand how users communicate according to the sentiment they send and receive from others.

### 3.3. User sentiment and social structure

Sentiment analysis models may struggle with a few characteristics of opinionated texts, such as (i) identifying sarcasm or irony, which are common in written language, (ii) adequately handling negations or contrasting statements, and (iii) managing domain-specific sentiments or jargon [72,73]. Therefore, the sentiment score obtained for a single tweet may provide unreliable information about the user’s generally expressed sentiment toward a topic.

To obtain a more robust indication of the users’ sentiment, the scores of all the tweets produced by one user were aggregated to obtain a single score (average of the scores obtained from all tweets by each user in the mutual mentions network), as exemplified in figure 13 in appendix A. Those users are highly active (given our process of constructing the mutual mentions network), therefore our users’ sentiments are averaged over a number of tweets, making it a robust measurement. The rescaled scores obtained with the AFINN lexicon are used to calculate the average sentiment in (of all tweets received) and out (of all tweets sent) of each user. In §3.5, these scores will form an important part of classifying users as Yes or No supporters.

**Figure 4.**
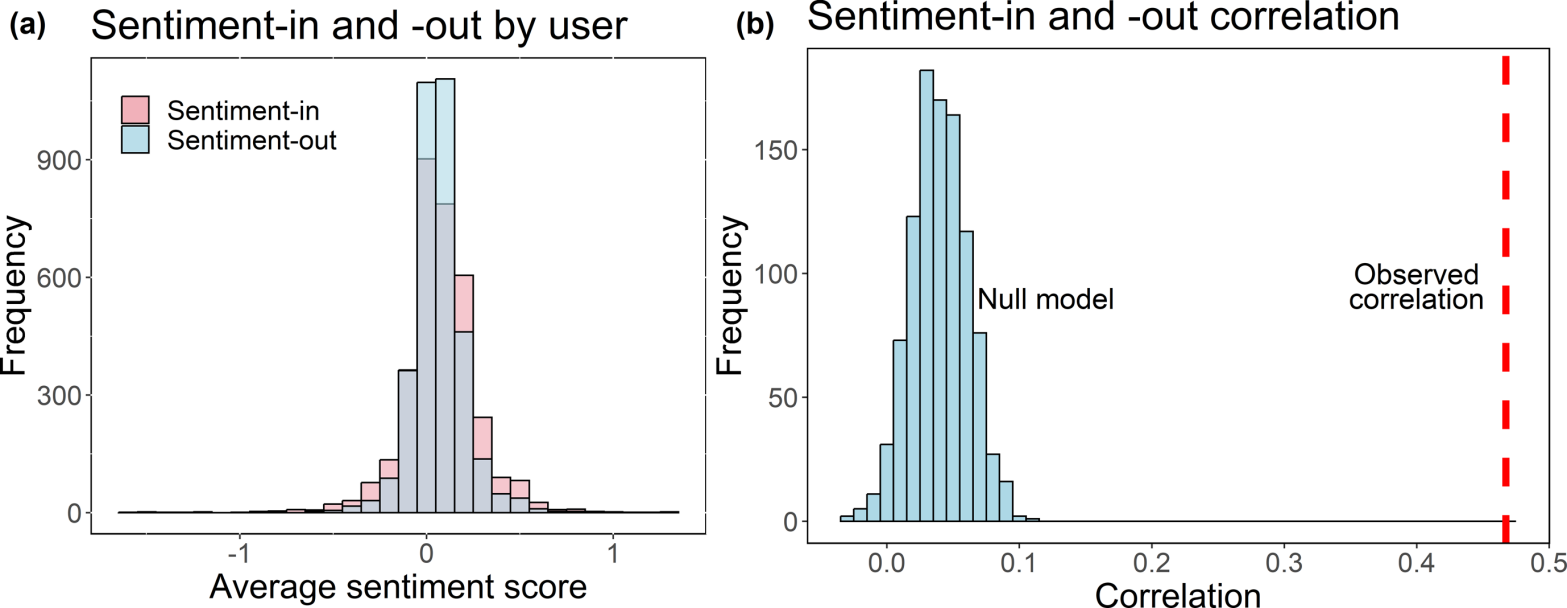
(*a*) Distribution of average sentiment-in by user in blue, and average sentiment-out by user in pink; (*b*) distribution of simulated correlation values between the sentiment-in and sentiment-out by user and the observed correlation. The blue bars are the resultant correlation distribution obtained after 1000 simulations, and the red dashed line represents the observed correlation.

Both users’ in-sentiment and out-sentiment scores distributions are centred on zero and slightly right skewed (figure 4*a*). Regarding the in-sentiment scores’ distribution obtained by the RT8 data, 93.76% of the users send tweets scoring between −0.4 and +0.4 on average, 71.79% of which are between −0.2 and +0.2. The out-sentiment distribution records 96.91% of the users scoring an average sentiment-out between −0.4 and +0.4, 83.93% of which are between −0.2 and 0.2 interval. Also, 65.75% of the users record a positive in-sentiment average score, while 70.66% of the users send positive tweets to others.

These results raise two main questions:

- Are user in- and out-sentiment scores correlated?
- Do users whose tweets have similar sentiments tend to be grouped in the network?

If the sentiments of the tweets that a user sends and receives are correlated, and users tend to cluster together with others that share similar sentiment, we could then consider sentiment alignment as a proxy for homophily among users. We can reasonably expect this as users with a similar disposition towards the referendum may communicate using similar language [9,74]. On a user level, the randomization tests analyse how strong the sentiment is as a signal, i.e. if the sentiment was very noisy, the correlation between the sentiment that a user sends and the sentiment they receive from neighbours would also be very noisy and therefore be a poor measure of similarity of connected users.

To answer (i), the correlation between users’ in- and out-sentiment is examined. The observed Pearson correlation between sentiment-in and sentiment-out score distributions is 0.4672, which indicates a moderate positive linear relationship between these two attributes [75,76]. To confirm that this correlation is not due to chance, a randomization procedure based on redistributing the sentiment of a user’s tweets is undertaken. The randomization procedure is as follows:

- Sample a sentiment score for each connection from the reciprocal network with replacement. Here, we use sampling with replacement to make sure the probability of selecting any particular sentiment score remains the same in future draws. This procedure also keeps the network topology intact.
- Calculate the average randomized in- and out-sentiment of each user.
- Calculate the correlation coefficient between the average randomized in- and out-sentiment distributions.
- Repeat steps (i)−(iii) 1000 times to create the null model for where the correlation coefficient could lie if there was no connection between the in- and the out-sentiment for each user.

Figure 4*b* shows the comparison of the resulting distribution of the correlation between the average randomized in- and out-sentiment scores after 1000 iterations of the randomization procedure with the observed correlation of sentiment-in and sentiment-out scores on the observed mutual mentions network for the Repeal the 8^th^ Referendum—0.47. The fact that the correlation obtained from the randomization is far away from the observed correlation indicates that there is a non-trivial correlation between the sentiment of what a user sends and receives. The observed correlation between sentiment-in and sentiment-out scores suggests that users may be more likely to be connected to other users with similar sentiment scores (meaning that they tend to use similar language about the referendum). On the network topology level, this is an indication that users may be grouped together according to the sentiment score, i.e. if the sentiment-in and sentiment-out of a user are correlated, this user is connected to others that send them similar sentiment as to what they send out to others.

Therefore, to assess if users tweeting similar sentiment tend to be grouped in the network, three class labels are created for users according to their sentiment—aggregate scores: above zero are ‘positive’, less than zero are ‘negative’ and scores equal to zero are ‘unknown’. We used these labels to find the fraction of connections between users of these classes. We denote the fraction of links between positive and positive users as *fpp*, the fraction of links between positive and negative users as *fpn*, between positive and unknown users as *fpu*, and so on. In total, there are nine types of links: *fuu*, *fup*, *fun*, *fnn*, *fnu*, *fnp*, *fpp*, *fpu* and *fpn*. We then randomize the class labels of each user by sampling from the observed distributions with replacement and recalculate the fraction of connections; we repeat this process 1000 times. As before, we compare the randomized distributions of the fractions with the observed fraction in our data.

**Figure 5.**
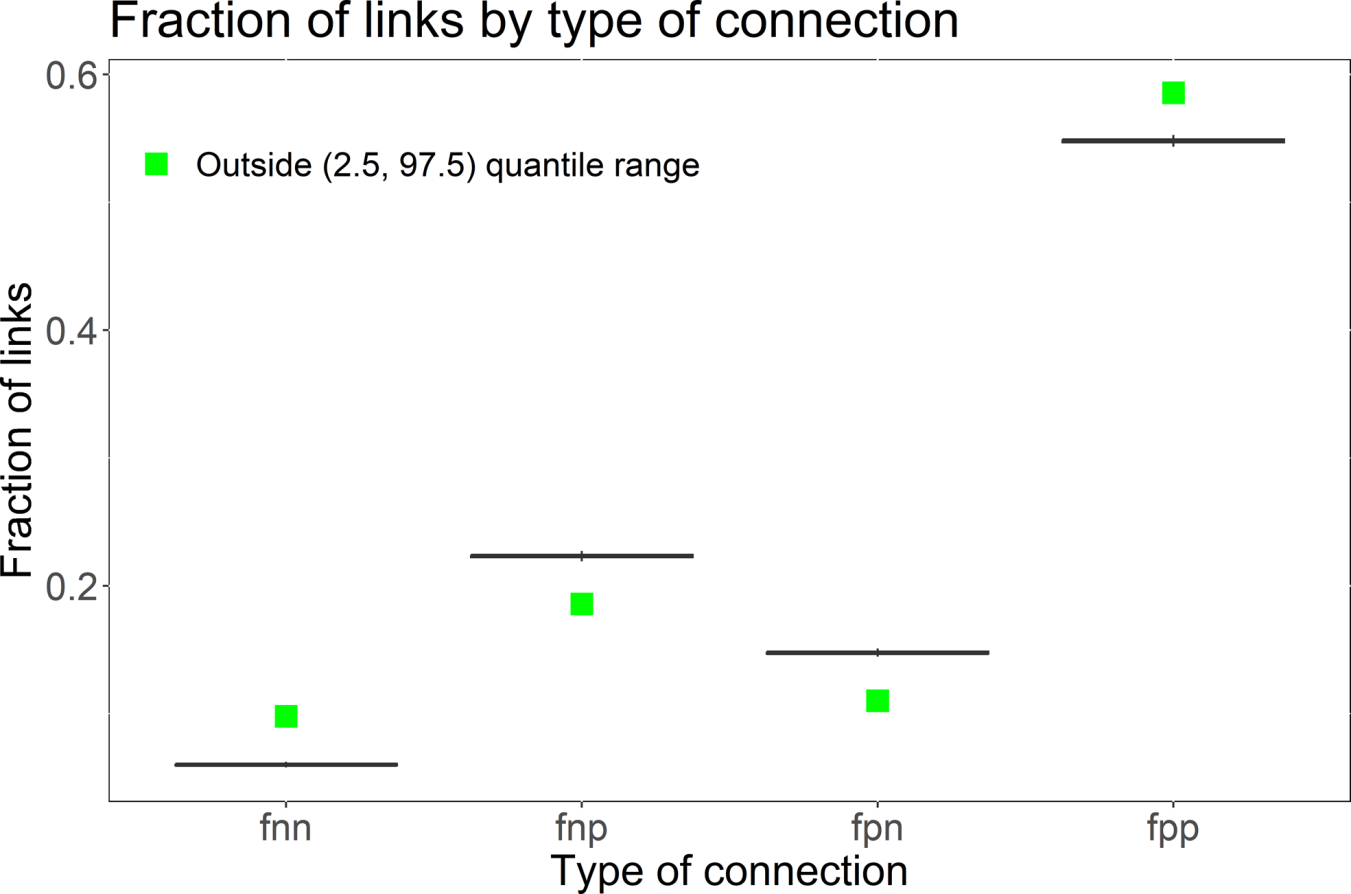
Results of the randomization test in the mutual mentions network. Green squares indicate that the observed fraction of connections falls outside the lower 2.5% and upper 97.5% quantiles of the randomized distribution (i.e. it is unlikely to arise by chance). We denote the fraction of links between negative and negative users as *fnn*, the fraction of links between negative and positive users as *fnp*, between positive and negative users as *fpn* and between positive and positive users as *fpp*.

We are interested primarily in the types of connections among positive and negative nodes, which are shown in figure 5. All the observed *fnn*, *fnp*, *fpn* and *fpp* lie outside the (2.5%,97.5%) quantile range, meaning that connections among negative and positive nodes do not occur at random. Moreover, the same types of nodes (*fnn* and *fpp*) have more links between each other than what is resultant from the random distribution, while opposite types of nodes (*fnp* and *fpn*) present smaller proportions than what would be expected at random. This is in agreement with the idea that users who show similar sentiments tend to be more connected than users who tweet opposing sentiments on the matter. Therefore, we could consider sentiment alignment as a proxy for homophily among users, i.e. ‘the tendency of nodes to connect to others who are similar on some variable’ [77], the variable being the aggregated sentiment score per user in our analysis.

We show that the relationships among users are not randomly distributed in the network. In fact, users who tweet similar sentiments tend to be grouped together. On the network topology, this may be interpreted as a proxy for community structure in the network, where there are distinct groups of users who share the characteristics of sending (and receiving) similar sentiments to other users. Therefore, we are interested in finding community structures in the network using the sentiment scores as a proxy for how close or far apart users are. In §3.4, we analyse whether users with similar aggregated sentiment scores are in the same network communities and can be clustered together.

### 3.4. Finding polarized communities

Polarization is commonly used to describe the division of society into groups that believe in opposite ideas. It has the potential to raise hostility and conflicts as well as occasional intergroup violence. However, it can also be a venue to challenge inequalities in societies and influence changes in policies and behaviours [78]. A referendum is a way to lead to policy changes; therefore, our aim in this section is to find out if there is a clear suggestion of polarization in the online discussion during the days leading to the referendum.

The results of the randomization process shown in figure 5 suggest a polarized environment. However, we can also use more standard methods to show polarized groups within the network. The tendency of people to create groups based on a shared characteristic is a common phenomenon in many social networks [54]. These groups—the network communities—can lead to a polarized environment in the context of a referendum, where people either vote in favour or against it [9]. In this section, we uncover communities based on the network structure and classify each community by observing the sentiment that users in each community send in the network. It is important to note here that our classification of users into communities is not given by the sentiment scores alone but by the network structure, where the weight of an outlink is the absolute value of the user’s average sentiment score. Here, we use the absolute value of the sentiment score for two reasons. (i) The community detection algorithms do not allow negative edge weights and (ii) the absolute value of the sentiment score can inform if users have a tendency to share more positive (or negative) language about the topic in discussion. As discussed in the previous section, users who share the same type of sentiment about the topic tend to group, and users who share extreme language (either positive or negative) tend to be weakly connected to the opposite extreme. Therefore, ‘extreme’ users (the ones sharing extreme language on average) tend to be apart from their ‘extreme’ opposites on the network topology level, and the absolute average sentiment score as link weight together with the network topology constitutes a strong structure to uncover polarized communities. Finally, we validate our classification with annotated data.

To check if users who share similar sentiments tend to be grouped together in the same network community and support our assumption that there exists polarization in this social network, we apply different community detection algorithms and analyse the partition found by the algorithm that performs the best. Table 4 in the appendix summarizes how well some commonly used community detection algorithms perform when taking the modularity as the parameter of optimization. Modularity measures the quality of each partition in comparison with a random configuration. Consider a network with N nodes and L links and a partition into $n_{c}$ communities, each community having $N_{c}$ nodes connected to each other by $L_{c}$ links, where $c=1,...,n_{c}$. The number of links within the community c is defined as $L_{c}$, and $k_{c}$ is the total degree of the nodes in the community c. To check if the local link density of the subgraphs defined by a partition differs from the expected density in a randomly wired network, we define the partition’s modularity by summing the modularity over all $n_{c}$ communities (equation (3.1)). The larger the value of M, the better the corresponding community structure [16]. By taking the whole network as a single community we obtain M=0, and if we assign each node of a network to a different community, the modularity is negative.


(3.1) $$\begin{array}{ccc} & M=\sum_{c=1}^{n_{c}}\left[\frac{L_{c}}{L}-{\left(\frac{k_{c}}{2L}\right)}^{2}\right]. & \end{array}$$

It should be noted that while modularity maximization methods have been shown to have some problems (e.g. resolution limit [79]), here we are looking for two communities (the Yes and No supporters) rather than an arbitrary number, and so modularity-based methods perform well given this constraint.

By following only the modularity optimization, the Leiden algorithm [80] performs best. However, the weighted Louvain [81] (using the absolute sentiment as the weight of a link) closely follows Leiden in modularity, and can find two, and only two, large communities, which are very distinct from each other and representative of the opposite sides of the discussion. Therefore, we have chosen to carry out our analysis with the two largest communities found by using the weighted Louvain method.

Table 3 is a summary of the properties of each community. We denote C1–C1 as the subgraph built with users in Community 1 only, i.e. effectively C1 only. Similarly, C2–C2 is effectively the C2. C1–C2 is the subgraph containing the links from C1 to C2, and C2–C1 is the subgraph containing the links originating in C2 and ending in C1. Community 1 (C1) is approximately six times the size of Community 2 (C2) in number of nodes (that is comparable to the number of nodes in the Yes and No communities observed for the Irish Marriage Referendum, seen in appendix D). Although C1 has a higher average out-degree than C2, C2 is more tightly connected as it presents a slightly higher average cluster coefficient and higher density. In contrast, both C1–C2 and C2–C1 present lower average out-degree and are less tightly connected than their counterparts since both average cluster coefficient and density are smaller. We also observe that users in C2 mention C1 more frequently than the opposite. This may be due to the fact that C2 is much smaller in number of nodes than C1.

**Table 3.** Summary table of community clusters.

	Nodes	Links	Avg. out-degree	Avg. cluster coeff.	Density
C1–C1	2954	110 351	18.373	0.376	0.006
C2–C2	463	5165	3.752	0.378	0.008
C1–C2	861	2401	1.777	0	0.002
C2–C1	897	4955	2.881	0	0.003

**Figure 6.**
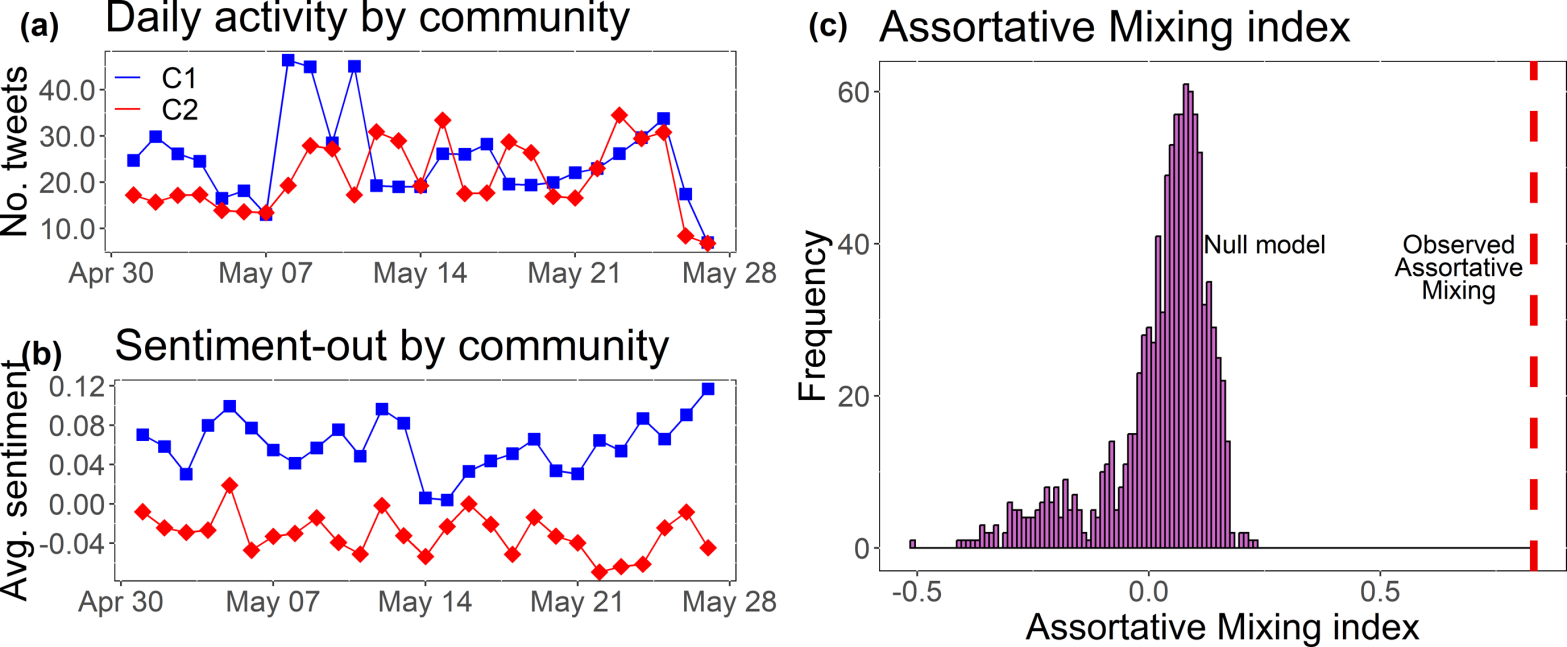
(*a*) Daily average number of tweets by community over time. C1 (Yes community) in blue and C2 (No community) in red. (*b*) Average sentiment-out of communities over time analysed. C1 presents a higher average sentiment-out over time than C2. (*c*) Monte Carlo simulation (histogram) of the assortative mixing index together with the observed assortative mixing index. The purple bars are the resultant correlation distribution obtained after 1000 simulations, and the red dashed line represents the observed index (0.83—high assortative mixing).

To classify the communities into the Yes community and the No community, we check the average sentiment-out of each of the communities over time (figure 6*b*). We classify C1 as the Yes community—it presents a higher average sentiment-out over time—and C2 as the No community, since it presents a lower average sentiment-out over time. Figure 6*a* shows that there is no significant difference between each community's activities over time. Figure 6*c* reassures that the assortative mixing in our network is significant. The simulation process is as follows:

(i) Randomly assign a community membership (1 or 2) to each user in the mutual mentions network with replacement. Here, we use sampling with replacement to make sure the probability of selecting either community membership remains the same in future draws. This procedure also keeps the network topology intact.

(ii) Calculate the assortative mixing index r for the network with new community assignments.


(3.2) $$\begin{array}{ccc} & r=\frac{\sum_{i}e_{ii}-\sum_{i}a_{i}b_{i}}{1-\sum_{i}a_{i}b_{i}}, & \end{array}$$

where $e_{ii}$ is the observed fraction of links that connect two nodes which both have value i, $a_{i}$ is the probability that a link has origin in a node with value i, and $b_{i}$ is the probability that a link has as destination a node with value i [82].

(iii) Repeat steps (i) and (ii) 1000 times to create the null model for where the assortative mixing index could lie if there was no connection between the network structure and the community assignment.

The observed assortative mixing (0.83) is high on a scale that ranges from −1 (disassortative) to 1 (complete assortativity) [82], while the simulations lie between (−0.3,0.2).

**Figure 7.**
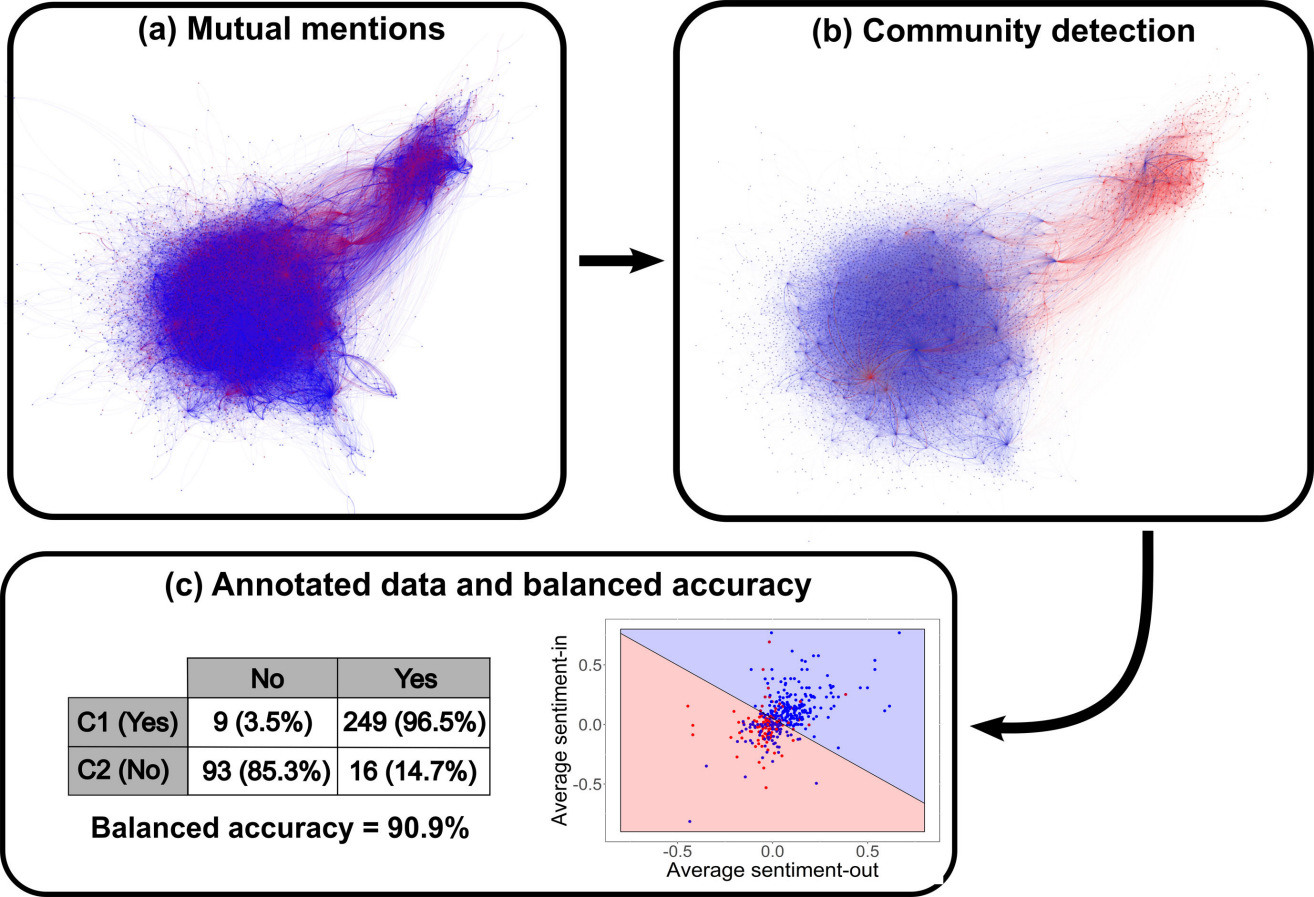
(*a*) Mutual mentions network representation with nodes and links coloured by the sentiment (positive or negative) of each individual; (*b*) mutual mentions network representation with nodes and links coloured by the community of each individual, after applying the Louvain community detection algorithm; (*c*) to check accuracy, we hand-classified over of the users in the mutual mentions network. The majority of the Yes supporters lie on top of the Yes community, and the opposite is also true. The balanced accuracy^2^ of our method of community identification is 90.9%.

Figure 7 shows how the community detection method accounts not only for the user sentiment scores (figure 7*a*) but also for the social structure. In figure 7*b*, the distinction between the two communities is clear and there is a suggestion of polarization, where each ideological community tends to be apart from each other, with little conversation in between. To determine the performance of our method of classification, we manually classify a stratified random sample of 10% of users in Community 1 and 10% of users in Community 2 as either Yes or No supporters. The manual classification is based on all tweets that the user being analysed sends to others in our network. If the language they use is indicative of their support for the Yes side of the debate, they are classified as a Yes voter, and likewise, a user that sends tweets suggesting support for the No side is classified as a No voter. We check this distribution against the community assignment, which can be seen in figure 7*c*. This shows that the majority of manually classified No supporters are in the No Community (C2) and the majority of the Yes supporters lie in the Yes Community (C1). From the confusion matrix, we have a good agreement between our method of classification and reality (based on data annotation). The overall balanced accuracy of the method is 90.9%, which is considered very good [83,84]. This result is more accurate than the one obtained by using a combination of the mentions and the followership networks (appendix C), and more accurate than the method using hashtags for user classification (appendix B).

To summarize our classification method, the following steps are needed:

- Build a conversation network among highly active users.
- Add weights for links between users where Weight = |Averaged out-sentiment of the node where the link starts|.
- Apply well-understood community classification methods on the network, using weights when allowed.
- Check which classification method returns the best results.
- (If needed) Apply a clustering method (e.g. k-means), where the parameters for clustering are the average in-sentiment and average out-sentiment of users, to cluster communities together. Appendix D shows the classification of the Irish Marriage Referendum network, which needs this step.
- Classify each community as prone to the Yes side or the No side of the debate given the sentiment their users send to others.
- Validate the classification method with annotated data.

**Figure 8.**
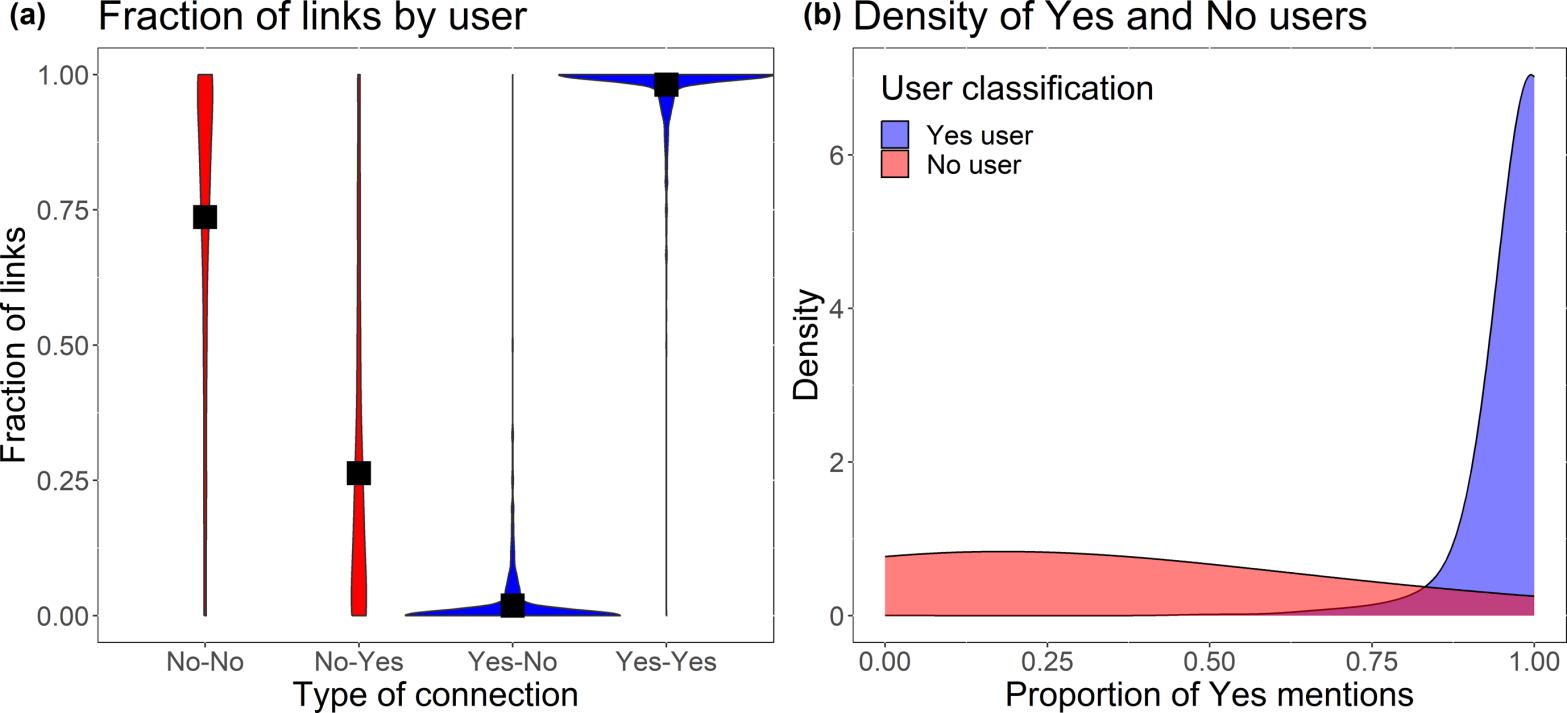
Fraction of connections between users in the two community clusters. (*a*) Violin plot for the distribution of types of links by user, aggregated by community type. The black squares represent the mean for each distribution. (*b*) Density plot for the distribution of Yes links by user, aggregated by community type.

To reinforce our point that communities in the Repeal the 8^th^ mentions mutual network are polarized, figure 8*a* shows the distribution of fractions of types of links by each user in the network. We observe that users tend to communicate with others in the same community and interact less with users in the opposite community. Figure 8*b* shows that it is unlikely that Yes users will mention a No user (as very few of them have links to No users). The opposite is still true, but to a lesser extent as there is a small number of No users who have links to the Yes side of the referendum. Given that we know, with a high degree of accuracy, the community structure and also how communities share information, we can analyse how information spreads inside and between communities. In the following section, we do this by reconstructing the cascades of information diffusion.

### 3.5. Information diffusion within and cross-community

Information diffusion can be represented as a cascade of events. A cascade allows us to trace the pattern of diffusion from the user that initiated the process (the seed) through a population [13]. It has been hypothesized by some that cascade behaviours are hard to predict [85]. However, insights as to how the network’s structure impacts the spreading of content and the mechanism by which it spreads can still be obtained [86–88]. We are particularly interested in how information spreads within and between communities [33].

To this end, we employ a popular cascade reconstruction method proposed by Goel *et al*. [13], in which they use the temporal sequence of retweets and the network structure to reconstruct the likely cascade structure. Even though this is a mechanistic rule-based method, it attained very reasonable performance in the original paper and in a previous application [36]. The process can be broken down into four steps: (i) bucketing Retweet IDs, (ii) ordering chronologically, (iii) parent attribution, and (iv) cascade ID assignment.

Following this process, we first compare every tweet in our collected dataset (but excluding tweets without mentions) to create Retweet IDs for each unique cleaned text (lower case, without mentions and hashtags), then order the tweets in the Retweet IDs according to the time they were posted. There are 114739 original tweets that were never retweeted. Of those, 29108 (25.37%) were written by Yes users, 6305 (5.49%) were tweeted by No supporters, and 79326 (69.14%) by users who were not assigned an ideological community.

**Figure 9.**
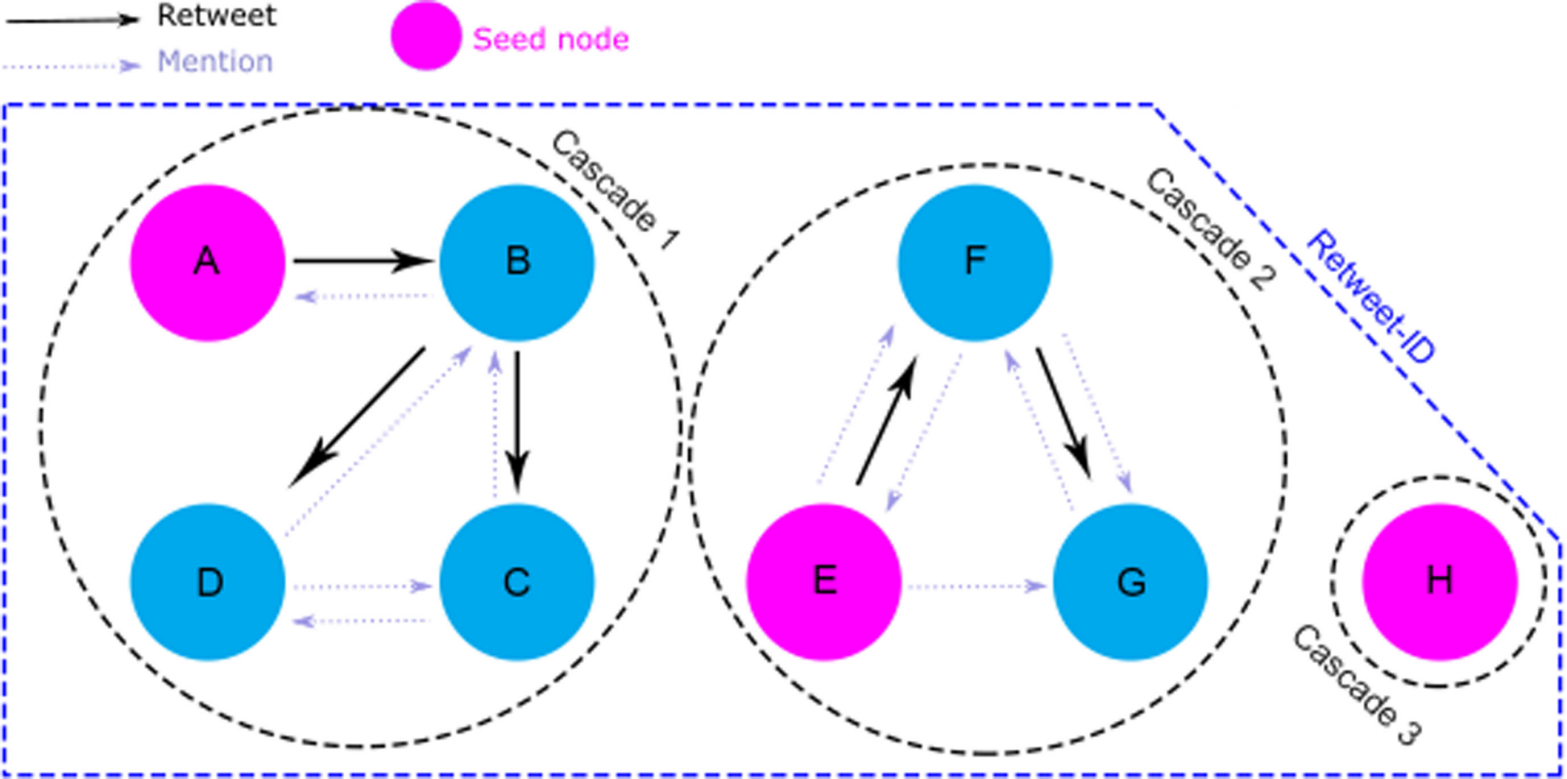
Method of finding parents and children using mentions in the network. Nodes are named according to the time where they (re)tweeted the content, i.e. A tweeted the content first in our timeline, while H was the last one to tweet that text. Pink nodes are the seeds of their cascades. Dashed grey arrows specify who mentions whom in the network, and full black arrows show the parenting assignment.

**Figure 10.**
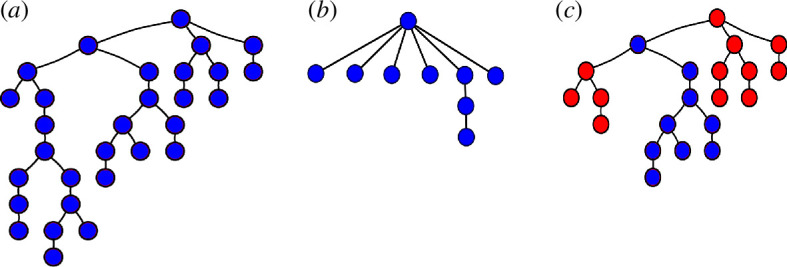
Examples of cascade trees built using the mentions network. Users in the Yes community are represented as blue nodes, while those in the No community are red. (*a*) A Yes-sided cascade that presents a viral behaviour, in which many users independently pass the information ahead. (*b*) A Yes-sided cascade that presents a broadcast behaviour, in which many users copy the content from one big source. (*c*) A cascade presenting a mix of broadcast and viral behaviours composed of both types of users (Yes and No supporters).

Next, we find who, among the user’s mentions (here, we again use the whole original dataset so that we avoid biases), is the most likely to have been retweeted from, for each retweet in each Retweet ID (figure 9). We assign a parent (A) to a mentioned user’s (B) retweet if they are the user who most recently could have introduced the content into B’s timeline (i.e. A tweeted the same content before B and B mentions A at some point in our network). If no such mention exists, we treat B as a root of an independent cascade, i.e. they posted that content independently, starting a new tree (e.g. users E and H in figure 9). From the original 15340 Retweet IDs containing at least two tweets each (the original tweet and at least one retweet), we retrieved 24983 cascades when applying the method of parent–children attribution. Examples of trees retrieved from this process are shown in figure 10, where blue nodes are users in the Yes community and red nodes are users in the No community.

**Figure 11.**
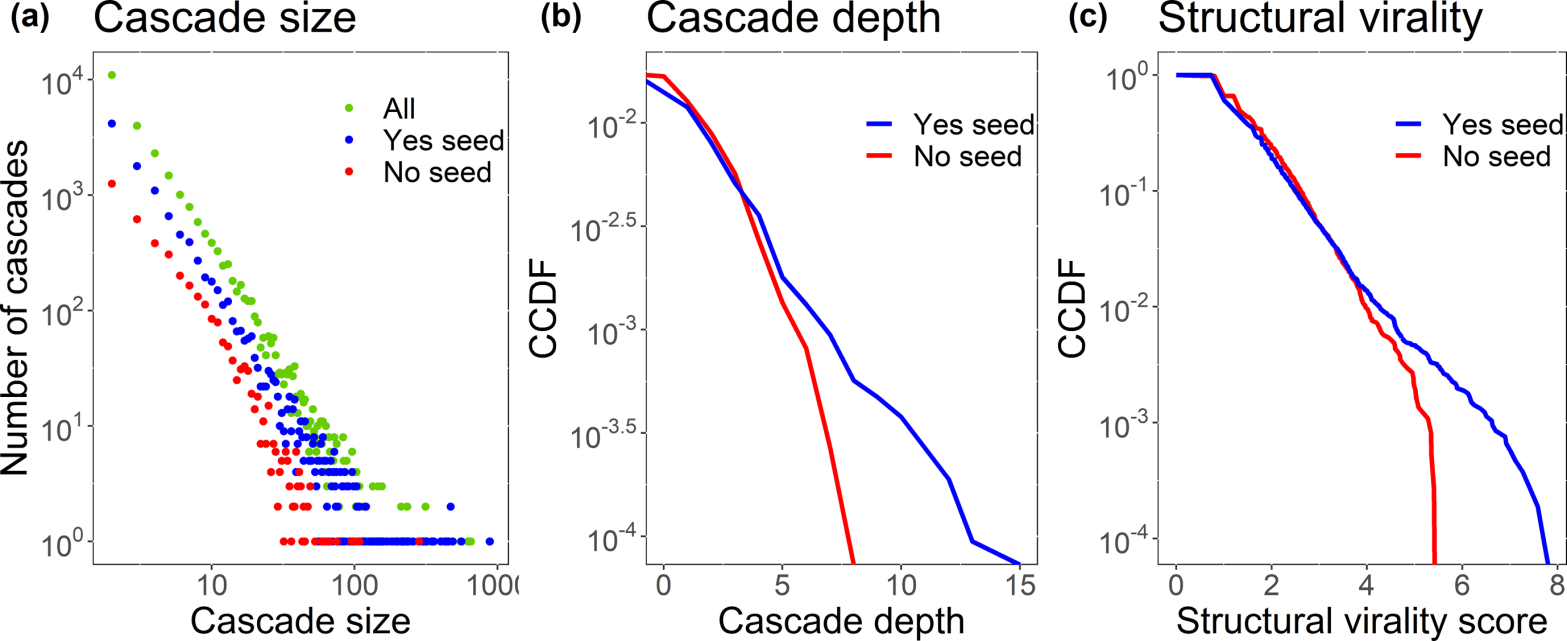
(*a*) Distribution of cascades sizes by seed community. (*b*) Complementary cumulative distribution function (CCDF) of maximum cascade depth by seed community. (*c*) CCDF of structural virality scores by seed community.

To analyse how the information spreads across the same ideological community and across different communities, we classify the cascade trees as Yes seeded or No seeded according to which community the first user who tweeted the content (the seed) belongs to. Figure 11 shows that the distribution of cascade sizes is heavy-tailed, that is, a large number of cascades are small (from two users), while only a small number of cascades are large—which complies with previous works [13–15,89]. Of the 24983 cascades retrieved (figure 11*a*, green curve), 10631 (42.5%) are Yes seeded (figure 11, blue curves) and 3774 (15.1%) are No seeded (figure 11, red curves). The remaining (42.3%) have seeds unclassified into communities—these could be unknown or could belong to one of the ideological communities but were filtered out when we chose to analyse only the strongest connected component of the mutual network and the two largest communities uncovered by the weighted Louvain method. The largest Yes-seeded cascade contains 884 users, and the largest No-seeded cascade contains 283 users. The disparity between the sizes of cascades may be due to the fact that there are fewer No supporters than Yes supporters in our network and also because the Yes-seeded cascades are more abundant than the No-seeded ones, therefore we have a smaller probability of finding large cascades amidst the No-seeded cascades. Depths and virality reach greater values on the Yes-seeded cascades (figure 11*b,c*). The Yes-seeded cascades reach depths as high as 15, while the No-seeded cascades do not surpass eight steps—again, this might be an effect of the disparity between the number of each type of cascade. The Yes-seeded cascades present slightly more viral behaviour (maximum virality of 7.8) in comparison with the No-seeded cascades (which reach virality of 5.4 the highest). The distributions observed for cascade size and cascade depth are comparable to the ones found in previous works on social media networks; however, the structural virality is slightly lower when compared with other online discussions [13–15], suggesting a more broadcast behaviour—many users sharing information from a small amount of key users in the discussion. See appendix E for more details on cascade scores.

**Figure 12.**
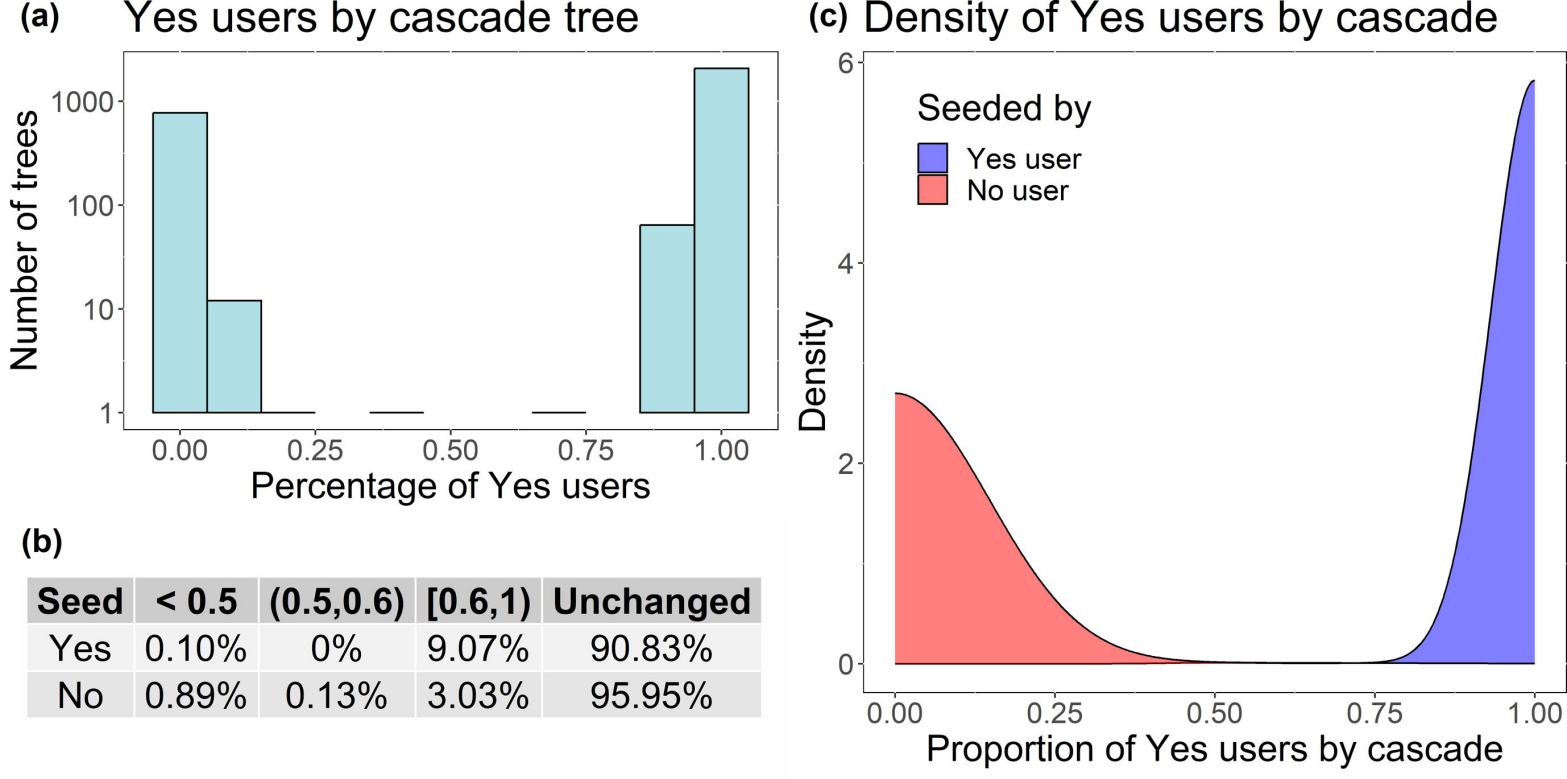
(*a*) Proportion of Yes users by cascade tree (of the cascades that have 10 or more classified users). Note that the *y*-axis is on a log-scale. (*b*) Behaviour of information diffusion by seed community (of the cascades that have 10 or more classified users). (*c*) Density of Yes users by cascade tree, coloured by cascade seed type (of all cascades).

**Figure 13.**
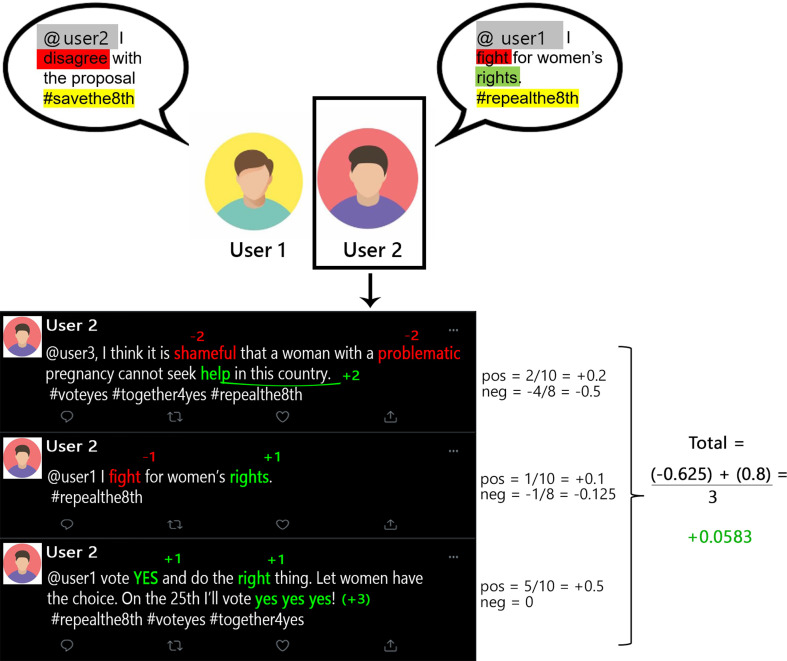
Example of how the mentions network was built and how we assign sentiment scores. Words are detected as positive (green) or negative (red) and given a score. The zoom in User 2’s tweets exemplifies how the rescaling and averaging were done. In this example, we consider that the highest positive score in a single tweet in the network is +50, and the lowest negative score in a single tweet in the network is −40. Note: the texts and usernames in this example are merely illustrative.

To determine how information spreads between and inside communities, of the 2918 cascades that have 10 or more classified users—the ones classified into one community or the other—only two (0.07%) have a percentage of Yes users between (0.25,0.75), which means that the majority of the cascades tend to present 75% or more users of the same ideological community (figure 12*a*). Or, in other words, information tends to diffuse inside communities, and less frequently between communities. We also observe (figure 12*b*) that 90.83% of the information that starts with a Yes seed remains in the Yes community, and over 95% of the information that starts with a No seed remains in the No community. The table shows the proportion of cascades that have less than 50% of the users in the same community as the seed (when we consider that the community where the information spread changed) between 50% and 60%, between 60% and 100%, and the proportion of cascades where all users are in the same community as the seed (Unchanged). It shows that only 0.10% of the cascades starting with a Yes user entered the No community and only 0.89% of the cascades starting on the No side spread to the Yes side of the discussion. Figure 12*c* shows the proportion of Yes users by cascade grouped by the type of seed of each cascade, now considering every cascade retrieved. We observe that the majority of No-seeded cascades present a low density of Yes users, while Yes-seeded cascades tend to have a high density of Yes users.

Therefore, we show that information tends to spread mainly within the same ideological community, and less frequently we observe information diffusion between the opposite ideological communities. Those results are comparable to the ones obtained by retrieving cascades using the followership network (appendix C)—which is known to be successful [13,14]—if not more interesting as it avoided splitting up cascades into smaller ones when a parent was not found. We observe that users do not always follow people they retweet from, but they probably interact with them at some point, be it in a reply or a mention of the content they post. Also, the mentions network tends to have more complete information in the sense that the followership is recovered only for the exact time when the data are being mined, which might take place years after the phenomenon we are studying occurred—as in our case, a 4-year gap—and, so, users might have stopped following others or might have deleted their accounts (in which case they would still be present in our mentions network, as mentions are not deleted by deletion of accounts).

We therefore show that the vast amount of information shared between two communities never leaves the host community; in effect there is an echo chamber of information. This also stands in contrast to figure 8*a*, where we noted that there was indeed a group of users who mentioned users in the other community (No did mention Yes), but these interactions did not result in retweets.

## 4. Discussion and conclusion

The understanding of social interactions, polarization of ideas, and the spread of information play an important role in society. We used Twitter data to identify opposing sides of the [43] debate and to observe how information spreads between these groups in our current polarized climate. We built a sentiment-based mentions network from the tweets concerning the debate to detect polarized communities—communities dominated by one type of user (Yes or No supporter)—with high balanced accuracy (90.9%). We showed that we were able to do it without the need for the followership network, which requires a time-consuming gathering process, and by using this simpler approach we achieved higher accuracy than previous work [9].

These communities and over 31000 retweets formed the basis of our analysis of information diffusion, which showed that information tends to spread heavily inside the same ideological community and less frequently between communities. This means that users tend to communicate mainly with those with whom they share an ideology and less with people who have opposing opinions regarding the abortion debate in Ireland. This provides a valuable methodology for extracting and studying information diffusion on large networks by isolating ideologically polarized groups and exploring the propagation of information within and between these groups. Once again, we did not make use of the followership network as seen in previous studies [13–15]. Our results show that by using the conversation network instead of the followership network for parenting attribution, we avoid splitting the Retweet-ID cascades into many smaller cascades by not finding followership links between users.

Some of the limitations of our analysis are due to the data gathering process, as we may not be able to retrieve all the original data regarding the referendum. However, the network that we created is large, well-connected and robust for our purposes. Another concern with the data gathering method is the recent changes to the Twitter API, as Twitter no longer gives researchers access to data via their academic API. Therefore, trying to collect Twitter data might incur unwanted extra expenses. Given that our method used significantly less data than previously published methods to arrive at nodal stances, with no loss in accuracy, this would be a preferred method to be used for node classification and information cascade building. Also, as we have seen, a sentiment lexicon might struggle to catch all the nuances of double negatives and irony [69]. However, we mitigated this weakness by (i) aggregating on a user-level so that if a user has a few sarcastic tweets, the majority of their tweets still have straightforward language, which prevails when aggregated and (ii) by performing multiple randomization tests to show that sentiment does indeed provide a signal that helps explain the network topology, where the sentiment that a user sends and receives is highly correlated, along with the connectivity patterns between Yes and No supporters. Finally, the use of a community detection method that may have its own limitations [79] proved to provide a valuable and accurate clustering of the users into two polarized groups. Another limitation that is worth discussing is the possibility of off-channel diffusion of information, which is not captured by our cascade inference method. The same piece of content might spread via other channels such as Facebook and YouTube, and two users who are connected on other platforms might independently post the same content on Twitter after seeing it on other platforms, and in this case, our method mistakenly treats a single diffusion tree as two disjoint events [13].

In future work, we plan to show how the empirical cascades compare with models of information diffusion. Given that we have found that information rarely spreads between communities on this topic, we are interested in finding (i) which of the popular models of information diffusion best capture this spread and (ii) whether the network structure sufficiently mediates the observed spread of information between the communities or whether we require information on community-specific content-spreading ‘attractiveness’. We will also use centrality measures to find the most influential users in our polarized environment and assess their performance when applied to a polarized social network. Furthermore, we would like to explore other methods for node classification in future work using machine learning algorithms, such as knowledge graphs and text embedding, and potentially extend our initial classification of very active nodes to more peripheral nodes in the discussion using label propagation. We would also like to explore other methods of cascade inference in the future, such as NetInf [90] and other statistically based methods, and assess how well these methods match each other on empirical datasets and how well they recreate known cascade structures from simulated data.

## Figures and Tables

**Figure 14.**
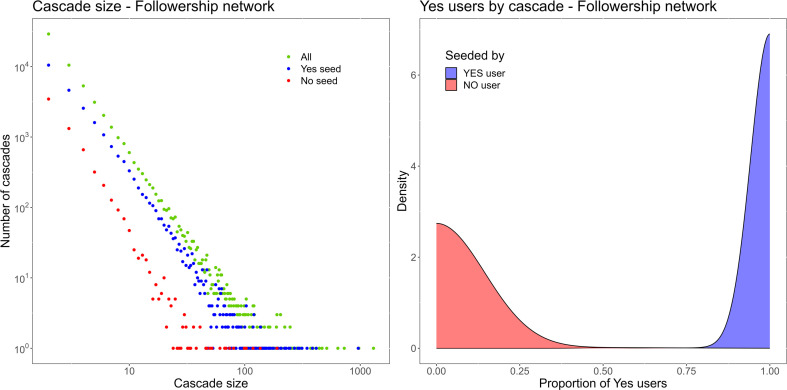
(*a*) Distribution of cascade sizes by seed community. (*b*) Density of Yes users by cascade tree, coloured by cascade seed type.

**Figure 15.**
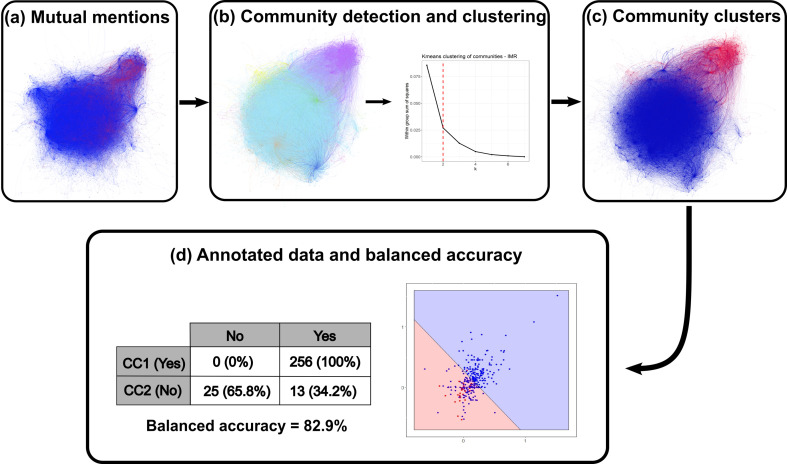
The community clustering process for the Irish Marriage Referendum mutual mentions network. (*a*) The mentions network before applying the community detection method (weighted Louvain), nodes and links coloured by sentiment (positive or negative). (*b*) Network visualization with nodes and links coloured by the initial eight community memberships resulting from the community detection method; k-means clustering of detected communities where the parameters are the average sentiment-in and sentiment-out of each community. The optimal number of clusters is two. (*c*) How the initial eight communities are clustered into two final community clusters, which we nominate ‘Yes community cluster’ and ‘No community cluster’. (*d*) When analysing annotated data for side of the debate support, the majority of the Yes supporters lie on top of the Yes community cluster, and the opposite is also true. The balanced accuracy of our method of community identification is 82.9%.

**Figure 16.**
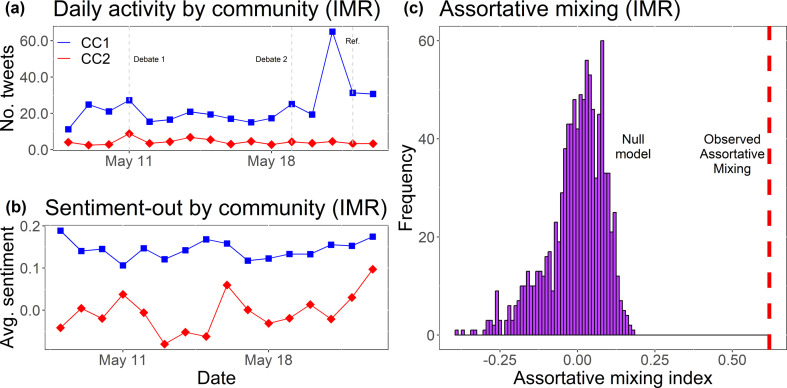
(*a*) Activity of the community clusters in the Irish Marriage Referendum discussion. The No community cluster has a peak (still weaker than the Yes community cluster) around the first televised debate, while the Yes CC has its peak of activity and is much more active than the No CC days before the Referendum day, when the majority voted Yes. (*b*) Sentiment-out of the community clusters in the Irish Marriage Referendum. Corroborating with the nomenclature we gave to the community clusters, the Yes CC (CC1) is constantly positive on average over time, while the No CC (CC2) is mostly negative or neutral on average over the time analysed, only leaning to a positive language after the referendum. (*c*) Monte Carlo simulation (histogram) of the assortative mixing index together with the observed assortative mixing index. The purple bars are the resultant assortative mixing index distribution obtained after 1000 simulations, and the red dashed line represents the observed index of 0.62.

**Figure 17.**
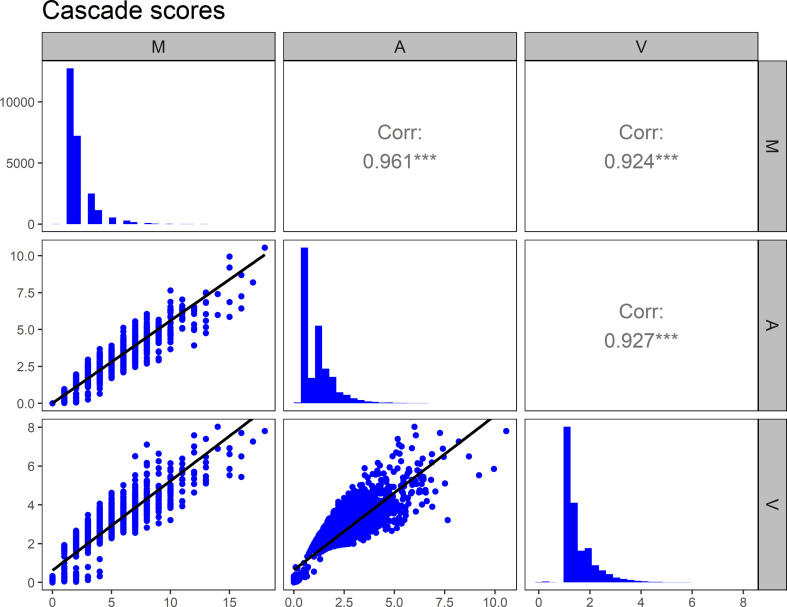
Pearson correlation, distribution plots and scatter plots of pairs of cascade scores. There is a statistically significant linear correlation between all measures presented above (p<0.001 in all three cases).

## Data Availability

Pseudo-anonymized data used in this study are available in github at https://github.com/caroline-pena/Repeal_the_8th_data. It contains the full mention network structure, the community memberships, and the retweet IDs, which will allow users to create their own cascades from the data. It also contains a file with the original tweet IDs. The tweet IDs allow one to gather the original tweets by using the Twitter API.

## Appendix A. Extra details

### A.1. Mentions network creation—an illustrative example

In this appendix, we provide an illustrative example of how the mentions network was created. In our example (figure 13), we consider two users: User 1 and User 2. Both User 1 and User 2 use tracked hashtags in their tweets, they are in the strongest connected component of the network, and they mention each other at least once. Therefore, their tweets concerning the Repeal the 8^th^ Referendum are in the mutual mentions network to be analysed. Each tweet is assigned a sentiment score by summing the rescaled positive with the rescaled negative scores. The rescaling depends on the maximum positive score and the maximum absolute negative score observed in a single tweet in the mutual mentions network. In our example, the maximum positive score is 50 and the maximum absolute negative score is 40 (which, divided by 5, the maximum allowed scores are 10 and 8, respectively). The resulting sentiment by the user is the average score of all their tweets in the network. A positive sentiment score means that, on average, the user tweets using more positive words than negative ones.

### A.2. Community detection algorithms

To find the best community detection algorithm for our case (§3.4), we compare some of the most used detection algorithms based on modularity. The results are shown in table 4. We call ‘significant’ the communities which had 20 nodes or more, selecting only those to compose our final mutual mentions network. The table shows that although the Leiden [80] detection method achieved the highest modularity score, Louvain [81] follows it closely and retrieves only two large communities, which are shown to be very representative of the polarized communities we are looking for.

**Table 4.** Summary table of community detection algorithms. ‘Significant’ communities are the ones containing 20 or more nodes.

Algorithm	Modularity	Number of communities
Leiden	0.298	13 communities, 11 significant
Louvain	0.280	217 communities, 2 significant
Spinglass	0.273	18 communities, 8 significant
Fast greedy	0.246	523 communities, 7 significant
Walktrap	0.195	49 communities, 7 significant
Leading Eigen	0.191	5 communities, all significant
Infomap	0.071	552 communities, 31 significant
Label propag.	0.003	39 communities, one containing almost all nodes

## Appendix B. Hashtags: data collection and community classification method

This section is a further look on the Repeal the 8^th^’s hashtags scraping and dynamics.

### B.1. Data collection

For data collection, we first gathered tweets containing either #repealthe8th or #retainthe8th, allegedly used by opposite sides of the discussion. From the initial dataset, we observed that the users in our scraped dataset would also often use the hashtags #loveboth, #together4yes and #savethe8th, therefore, the second step in the data scraping also included these hashtags. This process added 441399 (64% of the total) tweets and 81024 (42.9% of the total) users to the dataset.

Table 5 shows the 15 most used hashtags in the 688945 tweets gathered and their representativeness when considering every unique hashtag in the dataset. Those 15 hashtags together account for 75% of all hashtags and #repealthe8th, #together4yes and #savethe8th account for 51.04% of all the hashtags in our dataset. Some hashtags in the list are not specific to the Repeal the 8^th^ Referendum, such as #hometovote, #voteyes, #repeal, #ireland, #yes and #voteno, therefore could not have been used to scrape more tweets. Those hashtags together represent 15.45% of all hashtags in the dataset. We therefore show that the chosen hashtags used to gather data on the Twitter API are representative of the referendum’s online discussion.

**Table 5.** The 15 most common hashtags in the Repeal the 8^th^ dataset and their representativeness when considering every unique hashtag in the scrapped tweets. Here, the percentage is normalized on the 15 hashtags only. The hashtags we used sum to 68.05% of the considered hashtags.

Hashtag	Number	Percentage
repealthe8th	248 107	41.95%
together4yes	112 134	18.96%
hometovote	66 168	11.19%
savethe8th	42 204	7.14%
8thref	24 102	4.07%
voteyes	22 998	3.89%
togetherforyes	18 326	3.10%
repeal	9579	1.62%
ireland	9106	1.54%
yes	7254	1.23%
voteno	6781	1.15%
referendum2018	6555	1.11%
loveboth	6292	1.06%
repealedthe8th	6104	1.03%
lovebothvoteno	5694	0.96%

### B.2. Using hashtags to classify users into polarized communities

Bruns *et al*., Conover *et al*. and Chen *et al*. [53,91,92] showed that hashtags can be used to classify Twitter users as taking part on specific discussions. Darwish [93] showed that those who supported and opposed (on Twitter) the confirmation of Kavanaugh to the US Supreme Court were generally using divergent hashtags. Recuero *et al*. [94] and Chagas *et al*. [95] used hashtags to classify users into one side of the debate being analysed. In our work, we showed that we can use a combination of the network structure and sentiment analysis to do that classification with a high balanced accuracy of 90.9%. The section aims to analyse how the hashtag classification performs compared with the ground truth—manually classified users—and to our own method.

To classify a user into either a Yes voter or a No voter by analysing hashtags, we firstly classify each tweet in our dataset into Yes or No depending on the hashtags contained in the text (here, we consider #repealthe8th and #together4yes as indicative of the Yes side, and #savethe8th, #loveboth and #retainthe8th as proxy for the No side). We then classify each user by the most frequent side of their tweets. However, it is possible (and even common) that a person uses different—sometimes opposite—hashtags in the same tweet and across their tweets. The hashtag #repealthe8th is often used as a neutral hashtag to the discussion, as well as by the Yes side. We also observe people using the hashtag #repealthe8th together with #savethe8th, #loveboth and #retainthe8th, allegedly No-side hashtags. Furthermore, people may use opposite hashtags to call attention to the opposite side of the debate.

Table 6 shows the confusion matrix between the hashtag-based classification and the manual classification as described in §3.4. The balanced accuracy by using this method of classification is 88.88%, a bit smaller than but comparable to the balanced accuracy of our method of classification. Table 7 shows the confusion matrix between our method of classification and the hashtag-based user classification. The balanced accuracy between the two methods is 87.78%, which is a good agreement. Therefore, we conclude that for this specific network, where we have hashtags that are indicative of the side of the debate, both classification using hashtags and our classification method using sentiment and the network structure are possible and accurate, with our method still outperforming the hashtag classification.

**Table 6.** Confusion matrix hashtag-based user classification and manual classification. The balanced accuracy is 88.88%.

Hashtag/manual	No	Yes
No	84 (91.3%)	8 (8.7%)
Yes	21 (13.5%)	134 (86.5%)

**Table 7.** Confusion matrix between our method of classification and the hashtag-based user classification. The balanced accuracy is 87.78%.

Membership/hashtag	No	Yes
C1 (Yes)	54 (4.00%)	1294 (96.00%)
C2 (No)	226 (79.58%)	58 (20.42%)

However, for the Irish Marriage Referendum dataset, it is not possible to use the hashtag classification, since the hashtags contained in that dataset are both neutral—not indicative of a side in the debate. As shown in appendix D, our method of classification performs well on that dataset, giving a balanced accuracy of 82.9%. Therefore, we show that our method of classification can be used in any polarized social media discussion and does not rely on hashtag classification.

## Appendix C. The method using the followership network

The follower list of users was used to create a network combining information from mentions and followership, as done by O’Sullivan *et al*. [9], and then compared the results with the ones obtained by using only the mentions network. It was also used to infer parents and children among the different users who tweeted the same text by following Goel *et al*.’s [13] information diffusion analysis method. We retrieved all users who compose the Repeal the 8^th^ dataset and, using the Twitter Academic Research API again, we gathered all users followed by each user. We retrieved the followers of 181225 (95.9%) out of the 188928 users in the mentions dataset. The number of links in the followership dataset is over 18 million. The difference between the number of users gathered for the followership dataset in comparison with the mentions dataset is due to the method used to retrieve followers from a user. It works only for present active accounts, i.e. if a user in the mentions dataset has deleted or deactivated their account, their followers cannot be retrieved. We also observe that 95.4% of the users have a list consisting of 1 to 500 followers after filtering by followers that used at least one of the tracked hashtags. The user with the largest number of followers using the tracked hashtags has 12465 of them.

### C.1. Community detection on the combined mentions + followership network

As done by O’Sullivan *et al*. [9], we combine the mentions and the followers networks in order to detect ideological communities. Therefore, we can use the results as a comparison for how our new method using only the mentions network performs. We start by applying different community detection methods to the mentions network and to the followers network separately. Once again, the Leiden method [80] achieved the highest modularity values—slightly higher than Louvain [81]—but when carrying out the analysis to distinguish polarized communities, Louvain outperforms, therefore we chose to apply Louvain (and the reliability on the comparison between methods is higher). The modularity values when using Louvain are 0.270 for the followers network and 0.288 for the mentions network. We then filtered the users by the ones belonging simultaneously to the first five largest communities in the followers network and the first nine largest communities in the mentions network (only the communities with 20 nodes or more).

We retrieve 39 combined communities by analysing the interactions among the communities in the mentions network and the communities in the followers network. We then apply k-means to cluster similar communities together (as described in appendix D) and uncover four community clusters, being three Yes supporters and one No supporter. By comparing the users classification with the annotated data we used in §3.4, our final balanced accuracy for this method using a combination of the followers and the mentions networks is 86.95%, lower than the 90.9% achieved by using only the mentions network. This discrepancy may be due to deletions of accounts (which impact the follower network), especially accounts of users who supported the No vote, where we observed the lower accuracy (53.69%), and due to the extra user filtering when combining both networks, which impacts the capacity of the community detection method to retrieve a reasonable community structure.

### C.2. Cascades inferred by using the followership network

Following Goel *et al*.’s [13] method of finding parenting, we use the followership network to check from whom a user is most likely to have retweeted among the people they follow. By using this method, we retrieve 57430 cascades from the 15340 Retweet IDs containing at least two tweets each. This shows that this method of parenting attribution does not perform as well as the method when using the mentions network (described in §3.5)—where we were able to split the Retweet IDs into only 24983 cascades—meaning that the process did not find suitable parents for a reasonable amount of retweets and considered a new cascade formation, even though the text was exactly the same. The results of the seeding analysis (figure 14) are comparable to the ones achieved by using the mentions network as the network of inference. There is a higher number of small cascades in comparison to the results shown in figure 11*a*, and this is due to the fact that we split the Retweet IDs into a larger number of smaller cascades when using the follower network for cascade inference. Figure 14*b*, however, shows the same qualitative results as those shown in figure 12*c*. This clarifies that independent of which network we use to infer cascades, the polarization is clearly affecting the way information spreads.

## Appendix D. The Irish Marriage Referendum

The same-sex marriage referendum in Ireland was held on 22 May 2015, and put Ireland in the history as the first country to approve same-sex marriage by popular vote [96]. The following text was inserted in the Irish Constitution:

Marriage may be contracted in accordance with law by two persons without distinction as to their sex. [11]

The Yes vote prevailed by 62 to 38% with a large 60.5% turnout in the referendum. In total, 1201607 people voted in favour with 734300 against, giving a majority of 467307 votes. Only one of the Irish counties rejected the same-sex marriage, and the Yes vote was particularly pronounced in Dublin [96].

The Irish Marriage Referendum data analysed in this work were collected by Sinnia, a data analytics company, using the Twitter Gnip Power-Track API which returns a complete dataset, not just a sample [13] as do other APIs, such as Twitter stream API [97]. Every tweet containing the hashtags #marref and #marriageref from 8 to 23 May 2015 (the day after the referendum) was collected.

As we did for the Repeal the 8^th^ analysis, we construct the mutual mentions network with an average sentiment score by user. We use that network to find polarized communities by using the weighed Louvain [81] algorithm, for which the modularity is 0.23. There are eight large (containing more than 20 nodes each) communities found by using the detection method, therefore we use k-means statistical clustering [98] to find a smaller number of final communities that could explain the network polarization. By applying k-means, we cluster the eight communities together into two community clusters (CCs) (figure 15*b*). The two final community clusters are well connected on the inside and less connected between each other (figure 15*c*), and when compared with the annotated data (manually classified users), we get good agreement (figure 15*d*), with a balanced accuracy of 82.9%, which is bigger than the balanced accuracy—81.0%—achieved previously [9] when using a combination of the mentions and the followership networks.

Following the polarized graph figure and the figure with the annotated data, we classify CC1 (blue) as the Yes community cluster and CC2 (red) as the No community cluster. Figure 16*b* corroborates with our classification by showing that CC1 presents a consistent positive average sentiment-out over time, and CC2 has a lower average sentiment-out over time. Figure 16*a* shows that the activity (average number of tweets per user) is higher for CC1, and after debate 2—days preceding the voting—the Yes community has a high peak, that is, Yes supporters tweeted a lot more, on average, than the No supporters on the days preceding the referendum. The observed assortative mixing index is greater and far away from the simulated ones (figure 16*c*), indicating that the assortative mixing does not occur due to chance alone; instead, it depends on the network structure. The observed correlation of 0.62 suggests a moderate to high assortative mixing uncovered by the community detection method.

We conclude that by using a simpler method of network creation and community detection when compared with O’Sullivan *et al*.’s [9] method, we get successful results with a higher balanced accuracy, indicating that our simpler method, which uses less data, works better for the Irish Marriage Referendum data, as well as for the Repeal the 8^th^ data, as discussed in previous sections.

## Appendix E. Cascade scores

A well-explored method to access how viral, or how diffuse a cascade tree (T) is, is the assignment of scores to it. Here we follow the discussion in Goel *et al*. [13] to measure the success of a cascade. We will discuss the results obtained by using maximum cascade depth [Disp-formula A5-E1](equation (E 1)), average cascade depth (equation (E 2)) and structural virality (equation (E 3)). The maximum cascade depth, the most intuitive method for assessing the ‘success’ of a cascade, is not able to distinguish between a viral behaviour and a large broadcast with just one, long, multi-generational branch [13]. The average cascade depth corrects for this issue. However, it fails if the information spreads through a long path from the root and then is broadcast out to a large group of adopters. In this case, the cascade would have a high average depth (since most adopters are far from the root) even though most adoptions are the result of a single influential node. The structural virality, otherwise, can deal with both matters but it is possible to construct hypothetical examples for which it might fail, according to its authors [13].

(E 1)
$$\begin{array}{ccc} & M(T)=\max_{i}\;d_{ij}, & \end{array}$$

where $d_{ij}$ denotes the shortest path between nodes i and j (the seed node) in a given cascade tree T.

(E 2)
$$\begin{array}{ccc} & A(T)=\frac{1}{n}\sum_{i}^{n}d_{ij}, & \end{array}$$

where j is the seed node and n is the number of nodes in a given cascade tree T.

(E 3)
$$\begin{array}{ccc} & V(T)=\frac{1}{n(n-1)}\sum_{i}^{n}\sum_{j}^{n}d_{ij}. & \end{array}$$

Figure 17 shows the resultant distributions of scores of our retrieved cascades. There is a high positive correlation between all pairs of scores, and the distributions have similar behaviour, i.e. all of them present a right (or positive) skew with mode 1.0 for the maximum cascade depth, and 0.5 for both average cascade depth and 1.0 for structural virality, a median of 1.0, 0.8 and 1.33, and mean values of 1.89, 1.06 and 1.49, respectively, meaning that the majority of the cascades present more of a broadcast behaviour (small scores). Figure 11 shows that the majority of cascades are small, with many presenting only one retweet (one user retweeted from the seed user). Those small cascades present low scores, bringing the median and the mean down.

^1^
At the time of this analysis, X was still known as Twitter, therefore we will be referring to it as Twitter in this article.
^2^
Balanced accuracy ‌is ‌a metric ‌used to ‌assess the performance ‌of a ‌classification ‌method when the ‌classes are ‌imbalanced.$Balanced\;accuracy=\frac{\left(True\;Yes\;users\;rate+True\;No\;users\;rate\right)}{2}.$

## References

[1] Cihon P, Yasseri T. 2016 A biased review of biases in Twitter studies on political collective action. Front. Phys. **4**, 34. (10.3389/fphy.2016.00034)

[2] Shewale R. Twitter Statistics in 2023 — (Facts after ‘X’ rebranding). See https://www.demandsage.com/twitter-statistics/#:~:text=Twitter%20has%20around%20450%20million,daily%20active%20users%20(mDAU (accessed 2 September 2023).

[3] Kemp S. Digital 2022: Ireland, February. See https://datareportal.com/reports/digital-2022-ireland#:~:text=Numbers%20published%20in%20Twitter%27s%20advertising,total%20population%20at%20the%20time (accessed 2 September 2023).

[4] Bestvater S, Shah S, River G, Smith A. 2022 Politics on Twitter: one-third of tweets from U.S. adults are political. Washington, DC: Pew Research Center.

[5] Flamino J *et al*. 2023 Political polarization of news media and influencers on Twitter in the 2016 and 2020 US presidential elections. Nat. Hum. Behav. **7**, 904–916. (10.1038/s41562-023-01550-8) 36914806 PMC10289895

[6] Grčar M, Cherepnalkoski D, Mozetič I, Kralj Novak P. 2017 Stance and influence of Twitter users regarding the Brexit referendum. Comput. Soc. Netw. **4**, 6. (10.1186/s40649-017-0042-6) 29266132 PMC5732609

[7] Del Vicario MD, Gaito S, Quattrociocchi W, Zignani M, Zollo F. 2017 News consumption during the Italian referendum: a cross-platform analysis on Facebook and Twitter. In 2017 IEEE Int. Conf. on Data Science and Advanced Analytics (DSAA), Tokyo, Japan, pp. 648–657. (10.1109/DSAA.2017.33)

[8] Furman I, Tunç A. 2020 The end of the Habermassian ideal? Political communication on Twitter during the 2017 Turkish constitutional referendum. Pol. Internet **12**, 311–331. (10.1002/poi3.218)

[9] O’Sullivan DJP, Garduño-Hernández G, Gleeson JP, Beguerisse-Díaz M. 2017 Integrating sentiment and social structure to determine preference alignments: the Irish Marriage Referendum. R. Soc. Open Sci. **4**, 170154. (10.1098/rsos.170154) 28791141 PMC5541536

[10] Barrett GM. 2020 Some reflections concerning referendums in Ireland. SSRN Journal (10.2139/ssrn.3746377)

[11] Murphy Y. 2016 The marriage equality referendum 2015. Irish Pol. Stud. **31**, 315–330. (10.1080/07907184.2016.1158162)

[12] Hunt K. 2019 Twitter, social movements, and claiming allies in abortion debates. J. Inf. Technol. Polit. **16**, 394–410. (10.1080/19331681.2019.1659901)

[13] Goel S, Anderson A, Hofman J, Watts DJ. 2016 The structural virality of online diffusion. Manage. Sci. **62**, 180–196. (10.1287/mnsc.2015.2158)

[14] Vosoughi S, Roy D, Aral S. 2018 The spread of true and false news online. Science **359**, 1146–1151. (10.1126/science.aap9559) 29590045

[15] Juul JL, Ugander J. 2021 Comparing information diffusion mechanisms by matching on cascade size. Proc. Natl Acad. Sci. USA **118**, e2100786118. (10.1073/pnas.2100786118) 34750252 PMC8609637

[16] Newman MEJ. 2003 The structure and function of complex networks. SIAM Rev. **45**, 167–256. (10.1137/S003614450342480)

[17] Taylor CE, Mantzaris AV, Garibay I. 2018 Exploring how homophily and accessibility can facilitate polarization in social networks. Inf. **9**, 325. (10.3390/info9120325)

[18] Thelwall M. 2017 The heart and soul of the web? Sentiment strength detection in the social web with Sentistrength. In Cyberemotions (ed. J Holyst), pp. 119–134. Understanding Complex Systems. Cham, Switzerland: Springer. (10.1007/978-3-319-43639-5_7)

[19] Ranco G, Aleksovski D, Caldarelli G, Grčar M, Mozetič I. 2015 The effects of Twitter sentiment on stock price returns. PLoS One **10**, e0138441. (10.1371/journal.pone.0138441) 26390434 PMC4577113

[20] Zheludev I, Smith R, Aste T. 2014 When can social media lead financial markets? Sci. Rep. **4**, 4213. (10.1038/srep04213) 24572909 PMC5379406

[21] Alamoodi AH *et al*. 2021 Sentiment analysis and its applications in fighting COVID-19 and infectious diseases: a systematic review. Expert Syst. Appl. **167**, 114155. (10.1016/j.eswa.2020.114155) 33139966 PMC7591875

[22] Crable E, Sena M. 2020 Exploring sentiment towards contact tracing. In Proc. of the Conf. on Information Systems Applied Research, Virtual, vol. 2167, p. 1508, Journal of Information Systems.

[23] Lim S, Tucker CS, Kumara S. 2017 An unsupervised machine learning model for discovering latent infectious diseases using social media data. J. Biomed. Inform. **66**, 82–94. (10.1016/j.jbi.2016.12.007) 28034788

[24] Sesagiri Raamkumar A, Tan SG, Wee HL. 2020 Measuring the outreach efforts of public health authorities and the public response on Facebook during the COVID-19 pandemic in early 2020: cross-country comparison. J. Med. Internet Res. **22**, e19334. (10.2196/19334) 32401219 PMC7238862

[25] Singh R, Singh R, Bhatia A. 2018 Sentiment analysis using machine learning technique to predict outbreaks and epidemics. Int. J. Adv. Sci. Res. **3**, 19–24. (10.21275/v5i4.nov162724)

[26] Bermingham A, Smeaton A. 2011 On using Twitter to monitor political sentiment and predict election results. In Proc. of the Workshop on Sentiment Analysis Where AI Meets Psychology, Chiang Mai, Thailand, pp. 2–10. Asian Federation of Natural Language Processing.

[27] Budiharto W, Meiliana M. 2018 Prediction and analysis of Indonesia Presidential election from Twitter using sentiment analysis. J. Big Data **5**, 1–10. (10.1186/s40537-018-0164-1)

[28] Ramteke J, Shah S, Godhia D, Shaikh A. 2016 Election result prediction using Twitter sentiment analysis. In 2016 Int. Conf. on Inventive Computation Technologies (ICICT), Coimbatore, India, vol. 1, pp. 1–5, IEEE. (10.1109/INVENTIVE.2016.7823280)

[29] Caetano JA, Lima HS, Santos MF, Marques-Neto HT. 2018 Using sentiment analysis to define Twitter political users’ classes and their homophily during the 2016 American presidential election. J. Internet Serv. Appl. **9**, 1–15. (10.1186/s13174-018-0089-0)

[30] De Choudhury M. 2011 Tie formation on Twitter: homophily and structure of egocentric networks. In 2011 IEEE 3rd Int. Conf. on Privacy, Security, Risk and Trust and 2011 IEEE 3d Int. Conf. on Social Computing, pp. 465–470. Boston, MA: IEEE. (10.1109/PASSAT/SocialCom.2011.177)

[31] Yuan G, Murukannaiah PK, Zhang Z, Singh MP. 2014 Exploiting sentiment homophily for link prediction. In Proc. of the 8th ACM Conf. on Recommender Systems, Foster City, Silicon Valley, CA, pp. 17–24. New York, NY: ACM. (10.1145/2645710.2645734)

[32] Thelwall M, Buckley K, Paltoglou G. 2012 Sentiment strength detection for the social web. J. Am. Soc. Inf. Sci. Technol. **63**, 163–173. (10.1002/asi.21662)

[33] Weng L, Menczer F, Ahn YY. 2013 Virality prediction and community structure in social networks. Sci. Rep. **3**, 2522. (10.1038/srep02522) 23982106 PMC3755286

[34] Centola D. 2010 The spread of behavior in an online social network experiment. Science **329**, 1194–1197. (10.1126/science.1185231) 20813952

[35] Keating LA, Gleeson JP, O’Sullivan DJP. 2022 Multitype branching process method for modeling complex contagion on clustered networks. Phys. Rev. E **105**, 034306. (10.1103/PhysRevE.105.034306) 35428098

[36] Gleeson JP, Onaga T, Fennell P, Cotter J, Burke R, O’Sullivan DJP. 2021 Branching process descriptions of information cascades on Twitter. J. Complex Netw. **8**. (10.1093/comnet/cnab002)

[37] Dow PA, Adamic L, Friggeri A. The anatomy of large Facebook cascades. In 7th Int. AAAI Conf. on Weblogs and Social Media, vol. **7**, pp. 145–154, PKP Publishing Services Network. (10.1609/icwsm.v7i1.14431)

[38] Sun E, Rosenn I, Marlow C, Lento T. 2009 Gesundheit! Modeling contagion through Facebook news feed. In 3rd Int. AAAI Conf. on Weblogs and Social Media, vol. **3**, pp. 146–153, PKP Publishing Services Network. (10.1609/icwsm.v3i1.13947)

[39] Deza A, Parikh D. 2015 Understanding image virality. In 2015 IEEE Conf. on Computer Vision and Pattern Recognition (CVPR), Boston, MA, pp. 1818–1826. IEEE. (10.1109/CVPR.2015.7298791)

[40] Guerini M, Staiano J, Albanese D. 2013 Exploring image virality in Google plus. In 2013 Int. Conf. on Social Computing, Alexandria, VA, pp. 671–678. IEEE. (10.1109/SocialCom.2013.101)

[41] Alonso López N, Bautista PS, Giacomelli F. 2021 Beyond challenges and viral dance moves: Tiktok as a vehicle for disinformation and fact-checking in Spain, Portugal, Brazil, and the USA. Anàlisi. Quad. Comun. Cult. **64**, 65–84. (10.5565/rev/analisi.3411)

[42] Anderson A, Huttenlocher D, Kleinberg J, Leskovec J, Tiwari M. 2015 Global diffusion via cascading invitations: structure, growth, and homophily. In Proc. of the 24th Int. Conf. on World Wide Web, Florence, Italy, pp. 66–76. ACM Digital Library. (10.1145/2736277.2741672)

[43] Pena C, MacCarron P, O’Sullivan DJP. 2024 Twitter data on the Irish Abortion Referendum of 2018. See https://github.com/caroline-pena/Repeal_the_8th_data (accessed 2 August 2024).

[44] Lapowsky I. 2013 Ev Williams on Twitter’s Early Years. See https://www.inc.com/issie-lapowsky/ev-williams-twitter-early-years.html (accessed 23 June 2021).

[45] Field L. 2018 The abortion referendum of 2018 and a timeline of abortion politics in Ireland to date. Irish Pol. Stud. **33**, 608–628. (10.1080/07907184.2018.1500461)

[46] BBC News. 2012 Woman dies after abortion request ‘refused’ at Galway hospital. See https://www.bbc.com/news/uk-northern-ireland-20321741 (accessed 18 August 2021).

[47] The Irish Times. 2012 Thousands attend Savita vigils around the country. See https://www.irishtimes.com/news/thousands-attend-savita-vigils-around-the-country-1.749469 (accessed 18 August 2021).

[48] BBC News. 2018 Eighth amendment repealed as Irish president signs bill into law. See https://www.bbc.com/news/world-europe-45568094 (accessed 18 August 2021).

[49] Twitter developer platform. 2021 Academic research product track. See https://developer.twitter.com/en/products/twitter-api/academic-research (accessed 18 August 2021).

[50] Charlton N, Singleton C, Greetham DV. 2016 In the mood: the dynamics of collective sentiments on Twitter. R. Soc. Open Sci. **3**, 160162. (10.1098/rsos.160162) 27429774 PMC4929909

[51] Grindrod P, Lee TE. 2016 Comparison of social structures within cities of very different sizes. R. Soc. Open Sci. **3**, 150526. (10.1098/rsos.150526) 26998323 PMC4785974

[52] Beguerisse-Díaz M, McLennan AK, Garduño-Hernández G, Barahona M, Ulijaszek SJ. 2017 The ‘who’ and ‘what’ of #diabetes on Twitter. Digit. Health **3**, 2055207616688841. (10.1177/2055207616688841) 29942579 PMC6001201

[53] Conover MD, Ratkiewicz J, Francisco M, Gonçalves B, Menczer F, Flammini A. 2011 Political polarization on Twitter. In 5th Int. AAAI Conf. on Weblogs and Social Media, Barcelona, Spain. AAAI. (10.1609/icwsm.v5i1.14126)

[54] Newman MEJ, Park J. 2003 Why social networks are different from other types of networks. Phys. Rev. E **68**, 036122. (10.1103/PhysRevE.68.036122) 14524847

[55] Agarwal A, Xie B, Vovsha I, Rambow O, Passonneau RJ. 2011 Sentiment analysis of Twitter data. In Proc. of the Workshop on Language in Social Media, pp. 30–38. Portland, USA: Association for Computational Linguistics. (10.5555/2021109)

[56] Chmiel A, Sienkiewicz J, Thelwall M, Paltoglou G, Buckley K, Kappas A, Hołyst JA. 2011 Collective emotions online and their influence on community life. PLoS One **6**, e22207. (10.1371/journal.pone.0022207) 21818302 PMC3144870

[57] Liu B. 2012 Sentiment analysis and opinion mining. Synth. Lect. on Hum. Lang. Technol. **5**, 1–167. (10.1007/978-3-031-02145-9)

[58] Loughran T, McDonald B. 2011 When is a liability not a liability? Textual analysis, dictionaries, and 10‐Ks. J. Finance **66**, 35–65. (10.1111/j.1540-6261.2010.01625.x)

[59] Mohammad SM, Turney PD. 2013 Crowdsourcing a word–emotion association lexicon. Comput. Intell. **29**, 436–465. (10.1111/j.1467-8640.2012.00460.x)

[60] Saini S, Punhani R, Bathla R, Shukla VK. 2019 Sentiment analysis on Twitter data using R. In 2019 Int. Conf. on Automation, Computational and Technology Management (ICACTM), London, UK, pp. 68–72. IEEE. (10.1109/ICACTM.2019.8776685)

[61] Apala KR, Jose M, Motnam S, Chan CC, Liszka KJ, de Gregorio F. 2013 Prediction of movies box office performance using social media. In 2013 IEEE/ACM Int. Conf. on Advances in Social Networks Analysis and Mining (ASONAM 2013). New York, NY: IEEE. (10.1145/2492517.2500232)

[62] Kim Y, Kang M, Jeong SR. 2018 Text mining and sentiment analysis for predicting box office success. KSII T. Internet Info. **12**. (10.3837/tiis.2018.08.030)

[63] Rajput P, Sapkal P, Sinha S. 2017 Box office revenue prediction using dual sentiment analysis. Int. J. Mach. Learn. Comput. **7**, 72–75. (10.18178/ijmlc.2017.7.4.623)

[64] Nagamma P, Pruthvi HR, Nisha KK, Shwetha NH. 2015 An improved sentiment analysis of online movie reviews based on clustering for box-office prediction. In 2015 Int. Conf. on Computing, Communication & Automation (ICCCA), Greater Noida, India, pp. 933–937. (10.1109/CCAA.2015.7148530)

[65] Spruce M, Arthur R, Williams HTP. 2020 Using social media to measure impacts of named storm events in the United Kingdom and Ireland. Meteorol. Appl. **27**, e1887. (10.1002/met.1887)

[66] Barkur G. 2020 Sentiment analysis of nationwide lockdown due to COVID 19 outbreak: evidence from India. Asian J. Psychiatr. **51**, 102089. (10.1016/j.ajp.2020.102089) 32305035 PMC7152888

[67] Valdez D, Ten Thij M, Bathina K, Rutter LA, Bollen J. 2020 Social media insights into US mental health during the COVID-19 pandemic: longitudinal analysis of Twitter data. J. Med. Internet Res. **22**, e21418. (10.2196/21418) 33284783 PMC7744146

[68] Alvarez R, Garcia D, Moreno Y, Schweitzer F. 2015 Sentiment cascades in the 15M movement. EPJ Data Sci. **4**, 1–13. (10.1140/epjds/s13688-015-0042-4)

[69] Silge J, Robinson D. 2017 Text mining with R: a tidy approach. See https://www.tidytextmining.com/sentiment.html.

[70] Nielsen FÅ. 2011 A new ANEW: evaluation of a word list for sentiment analysis in microblogs. arXiv. (10.48550/arXiv.1103.2903)

[71] Hernández Farias I, Buscaldi D, Sánchez BP. 2014 Iradabe: adapting English lexicons to the Italian sentiment polarity classification task. In First Italian Conference on Computational Linguistics (CLiC-it 2014) and the fourth International Workshop EVALITA, Pisa, Italy, pp. 75–80. (10.12871/CLICIT20141O)

[72] Serrano-Guerrero J, Olivas JA, Romero FP, Herrera-Viedma E. 2015 Sentiment analysis: a review and comparative analysis of web services. Inf. Sci. **311**, 18–38. (10.1016/j.ins.2015.03.040)

[73] Pang B, Lee L. 2008 Opinion mining and sentiment analysis. Norwell, MA: Now Foundations and Trends. (10.1561/9781601981516)

[74] Xia Y, Gronow A, Malkamäki A, Ylä-Anttila T, Keller B, Kivelä M. 2024 The Russian invasion of Ukraine selectively depolarized the Finnish NATO discussion on Twitter. EPJ Data Sci. **13**, 1. (10.1140/epjds/s13688-023-00441-2)

[75] Benesty J, Chen J, Huang Y, Cohen I. 2009 Pearson correlation coefficient. In Noise reduction in speech processing, pp. 1–4. Berlin, Germany: Springer. (Springer Topics in Signal Processing). (10.1007/978-3-642-00296-0_5)

[76] Taylor R. 1990 Interpretation of the correlation coefficient: a basic review. J. Diagn. Med. Sonogr. **6**, 35–39. (10.1177/875647939000600106)

[77] Ognyanova K. 2016 Network analysis and visualization with R and igraph. In NetSciX 2016 School of Code Workshop, Wroclaw, Poland. (10.1007/978-3-319-28361-6). https://kateto.net/netscix2016.html.

[78] Smith LGE, Thomas EF, Bliuc AM, McGarty C. 2024 Polarization is the psychological foundation of collective engagement. Commun. Psychol. **2**, 41. (10.1038/s44271-024-00089-2) 39242857 PMC11332107

[79] Fortunato S, Barthélemy M. 2007 Resolution limit in community detection. Proc. Natl Acad. Sci. USA **104**, 36–41. (10.1073/pnas.0605965104) 17190818 PMC1765466

[80] Traag VA, Waltman L, van Eck NJ. 2019 From Louvain to Leiden: guaranteeing well-connected communities. Sci. Rep. **9**, 5233. (10.1038/s41598-019-41695-z) 30914743 PMC6435756

[81] Blondel VD, Guillaume JL, Lambiotte R, Lefebvre E. 2008 Fast unfolding of communities in large networks. J. Stat. Mech. **2008**, P10008. (10.1088/1742-5468/2008/10/P10008)

[82] Coscia M. 2021 The atlas for the aspiring network scientist. arXiv. (10.48550/arXiv.2101.00863)

[83] Brodersen KH, Ong CS, Stephan KE, Buhmann JM. 2010 The balanced accuracy and its posterior distribution. In 2010 20th Int. Conf. on Pattern Recognition, Istanbul, Turkey, pp. 3121–3124. IEEE. (10.1109/ICPR.2010.764)

[84] Wei Q, Dunbrack Jr RL. 2013 The role of balanced training and testing data sets for binary classifiers in bioinformatics. PLoS ONE **8**, e67863. (10.1371/journal.pone.0067863) 23874456 PMC3706434

[85] Martin T, Hofman JM, Sharma A, Anderson A, Watts DJ. 2016 Exploring limits to prediction in complex social systems. In Proc. of the 25th Int. Conf. on World Wide Web, Montréal, Canada, pp. 683–694. Geneva, Switzerland: ACM. (10.1145/2872427.2883001)

[86] Keating LA, Gleeson JP, O’Sullivan DJP. 2023 A generating-function approach to modelling complex contagion on clustered networks with multi-type branching processes. J. Complex Netw. **11**, cnad042. (10.1093/comnet/cnad042)

[87] Dave K, Bhatt R, Varma V. Modelling action cascades in social networks. ICWSM **5**, 121–128. (10.1609/icwsm.v5i1.14092)

[88] Peters K, Buzna L, Helbing D. 2008 Modelling of cascading effects and efficient response to disaster spreading in complex networks. IJCIS **4**, 46. (10.1504/IJCIS.2008.016091)

[89] Lerman K. 2016 Information is not a virus, and other consequences of human cognitive limits. Fut. Internet **8**, 21. (10.3390/fi8020021)

[90] Gomez-Rodriguez M, Leskovec J, Krause A. 2012 Inferring networks of diffusion and influence. ACM Trans. Knowl. Discov. Data **5**, 1–37. (10.1145/2086737.2086741)

[91] Bruns A, Moon B, Münch F, Sadkowsky T. 2017 The Australian Twittersphere in 2016: mapping the follower/followee network. Soc. Media Soc. **3**, 2056305117748162. (10.1177/2056305117748162)

[92] Chen THY, Salloum A, Gronow A, Ylä-Anttila T, Kivelä M. 2021 Polarization of climate politics results from partisan sorting: evidence from Finnish Twittersphere. Glob. Environ. Change. **71**, 102348. (10.1016/j.gloenvcha.2021.102348)

[93] Darwish K. 2019 Quantifying polarization on Twitter: the Kavanaugh nomination. In Social Informatics: 11th Int. Conf., SocInfo 2019, Doha, Qatar, pp. 188–201. Cham, Switzerland: Springer. (10.1007/978-3-030-34971-4_13)

[94] Recuero R, Zago G, Bastos MT, Araújo R. 2015 Hashtags functions in the protests across Brazil. Sage Open **5**, 2158244015586000. (10.1177/2158244015586000)

[95] Chagas V, Carreiro R, Santos N, Popolin G. 2022 Far-right digital activism in polarized contexts: a comparative analysis of engagement in hashtag wars. Med. Commun. **10**, 42–55. (10.17645/mac.v10i4.5622)

[96] Caollai E, Hilliard Mark. Ireland becomes first country to approve same-sex marriage by popular vote. See https://www.irishtimes.com/news/politics/marriage-referendum/ireland-becomes-first-country-to-approve-same-sex-marriage-by-popular-vote-1.2223646.

[97] Morstatter F, Pfeffer J, Liu H, Carley KM. 2013 Is the sample good enough? Comparing data from Twitter’s streaming API with Twitter’s firehose. In 7th Int. AAAI Conf. on Weblogs and Social Media, Cambridge, MA, pp. 400–408. AAAI press. (10.1609/aimag.v39i4.2841)

[98] Lloyd S. 1982 Least squares quantization in PCM. IEEE Trans. Inf. Theory **28**, 129–137. (10.1109/TIT.1982.1056489)

